# Identification of common fungal extracellular membrane (CFEM) proteins in *Fusarium sacchari* that inhibit plant immunity and contribute to virulence

**DOI:** 10.1128/spectrum.01452-23

**Published:** 2023-11-14

**Authors:** Zhen Huang, Yuming Zhou, Huixue Li, Yixue Bao, Zhenzhen Duan, Caixia Wang, Charles A. Powell, Kai Wang, Qin Hu, Baoshan Chen, Jisen Zhang, Muqing Zhang, Wei Yao

**Affiliations:** 1 State Key Lab for Conservation and Utilization of Subtropical Agri-Biological Resources, Guangxi Key Lab of Sugarcane Biology, Guangxi University, Nanning, China; 2 IRREC-IFAS, University of Florida, Fort Pierce, Florida, USA; University of Minnesota Twin Cities, St. Paul, Minnesota, USA

**Keywords:** CFEM domain, pokkah boeng disease, *F. sacchari*, effector protein, sugarcane, pathogenicity

## Abstract

*Fusarium sacchari* is one of the primary pathogens causing pokkah boeng disease in sugarcane in China. Common fungal extracellular membrane (CFEM) domain-containing proteins only exist in fungi and play a significant role in the interaction of pathogens and plants. However, the CFEM proteins in *F. sacchari* were not identified and functionally resolved. Of the 20 FsCFEM proteins identified from the genome of *F. sacchari*, 16 FsCFEM genes were cloned, while 4 FsCFEM proteins (Fs06761, Fs08184, Fs10706, and Fs13617) were identified as effector proteins that could suppress the programmed cell death triggered by BCL2-associated X protein in *Nicotiana benthamiana*. Subcellular localization analysis revealed that these four effectors could enter plant cells and are mainly located in the cell membrane and nucleus. By the yeast YTK12 secretion system, the signal peptides of four CFEM proteins were functionally verified. The expression levels of these four effector proteins were significantly increased in the *F. sacchari*-infected sugarcane plants. Through fungal gene knockout, three genes (Fs06761, Fs08184, and Fs13617) were found to be important pathogenic factors for pathogens. Our results support that the FsCFEM effector proteins were virulence effectors and might be involved in regulating plant immunity.

**IMPORTANCE:**

Common fungal extracellular membrane (CFEM) domain-containing protein has long been considered an essential effector, playing a crucial role in the interaction of pathogens and plant. Strategies aimed at understanding the pathogenicity mechanism of *F. sacchari* are eagerly anticipated to ultimately end the spread of pokkah boeng disease. Twenty FsCFEM proteins in the genome of *F. sacchari* have been identified, and four FsCFEM effector proteins have been found to suppress BCL2-associated X protein-triggered programmed cell death in *N. benthamiana*. These four effector proteins have the ability to enter plant cells and inhibit plant immunity. Furthermore, the expression of these four FsCFEM effector proteins significantly increases during the infection stage, with the three of them playing an essential role in achieving full virulence. These study findings provide a direction toward further exploration of the immune response in sugarcane. By applying these discoveries, we can potentially control the spread of disease through techniques such as host-induced gene silencing.

## INTRODUCTION

Pokkah boeng disease (PBD) is one of the primary fungal diseases that cause the decline of sugarcane yield in the world (1). PBD is a highly destructive disease caused by *Fusarium* species complex, including *F. moniliforme*, *F. sacchari*, *F. verticillioides*, and *F. moniliforme* var. *subglutinans* (2 – 6). *F. sacchari* is one of the notable *Fusarium* species that caused sugarcane PBD in China (6), and the most recent PBD outbreak occurred in 2017 in Dehong, Yunnan province. PBD has caused 10%–40% yield loss in select commercial sugarcane varieties, thus considerably affecting China’s sugarcane industry (7).

Pathogenic fungi secrete effector proteins to regulate their interaction with host plants and promote infection or suppress the host immune defense response (8 – 11). Cysteine residues and N-terminal signal peptides are typically found in effector proteins, small proteins with fewer than 300 amino acids involved in exocrine secretion (12); reportedly, there is no transmembrane domain (13, 14). Effector proteins have dual biological activities and act as virulence factors or non-toxic factors that have been widely accepted and determined by the final interactions between the pathogen and its host (8, 15, 16). Therefore, the discovery of effector proteins and further understanding of their functional characteristics can help develop a new breeding strategy in sugarcane for PBD resistance.

Only fungi contain the common fungal extracellular membrane (CFEM) protein, and eight cysteine residues are conserved in this domain (17, 18). The CFEM domain is similar to epidermal growth factors (EGF). The EGF domain acts as secretin and the adhesion receptors in host–pathogen interactions (19). The first CFEM protein was identified as *Magnaporthe grisea* adenylate cyclase (MAC1) interacting protein-ACI1 (17, 20). To date, several new CFEM proteins have been identified from various fungi, including *F. graminearum*, *M. grisea*, *Candida albicans*, *Botrytis cinerea*, *Colletotrichum graminicola*, *Verticillium dahliae*, and *L. theobromae* (21 – 27). Some CFEM proteins play essential roles in maintaining intracellular iron content (25, 28), including CFEM proteins in *Candida glabrata*, particularly CgCcw14 and CgMam3 (29).

Other CFEM proteins play various roles in some pathogenicity-related physiological processes, as shown in previous functional studies. In *B. cinerea*, BcCFEM1 was involved in inducing plant necrosis in tobacco leaves by transient expression of the protein (30). BcCFEM1 was induced and expressed at a high level in the early stage of infected bean leaves (30). Targeted destruction of BcCFEM1 reduced the virulence, conidia, and stress resistance of *B. cinerea* (30). The virulence of *B. cinerea* ∆CFEM-Bcin07g03260 mutant was revealed in tomato (31). Of the 24 CgCFEM proteins from maize anthracnose pathogen *C. graminicola*, only four CgCFEM proteins inhibited programmed cell death (PCD) induced by the BCL2-associated X protein (BAX) in *N. benthamiana* (24). The CFEM protein Pth11 containing seven transmembrane domains is a crucial G protein-coupled receptor that necessarily corrects the development of attachment, attachment-like structure, and pathogenicity in *M. grisea* (32). Two CFEM-containing proteins in *Magnaporthe oryzae*, MoCDIP2 and MoCDIP11, have been determined to induce apoptosis in cells of non-host and host plants, *N. benthamiana* and rice, respectively (33). Gene knockout has been widely used to verify gene function (34 – 36). In *Colletotrichum gloeosporioides*, CgDN3 encodes a new pathogenicity determinant associated with the biotrophic phase of infection (10). In *Bipolaris sorokiniana*, the deletion of the CsSp1 gene notably attenuated the pathogenicity of the pathogen. CsSp1 was a virulence effector and involved in triggering host immunity (36). In *Fusarium graminearum*, the CFEM effector proteins were identified, and FgCFEM1, FgCFEM3, FgCFEM5, FgCFEM8, FgCFEM18, and FgCFEMn1 were core virulence genes but not FgCFEM7, FgCFEM10, FgCFEM11, FgCFEM16, and FgCFEM21 (22, 23). To date, however, the CFEM proteins from sugarcane PBD pathogens are not well defined, and their potential mechanisms of action remain largely unknown.

In this study, we investigated the genome sequence of *F. sacchari* and identified 20 FsCFEM proteins that we observed to be relevant to the immunity responses of tobacco, a non-host plant. Additionally, four FsCFEM effector proteins were identified to inhibit BAX-induced apoptosis. The signal peptides of four effector proteins were sufficient for the yeast secretion system. We also performed subcellular localization of these four genes in *N. benthamiana* and observed their expression patterns in pathogen-infected sugarcane plants. The respective deletion of three genes (Fs06761, Fs08184, and Fs13617) significantly reduced the virulence of *F. sacchari*. Overall, this study provides genetic resources to explore the interactions between *F. sacchari* and host/non-host plants, in addition to enriching insight on the pathogenic mechanisms of the fungus, which warrant future research.

## RESULTS

### Identification of CFEM domain-containing proteins in *F. sacchari*

**Fig 1:**
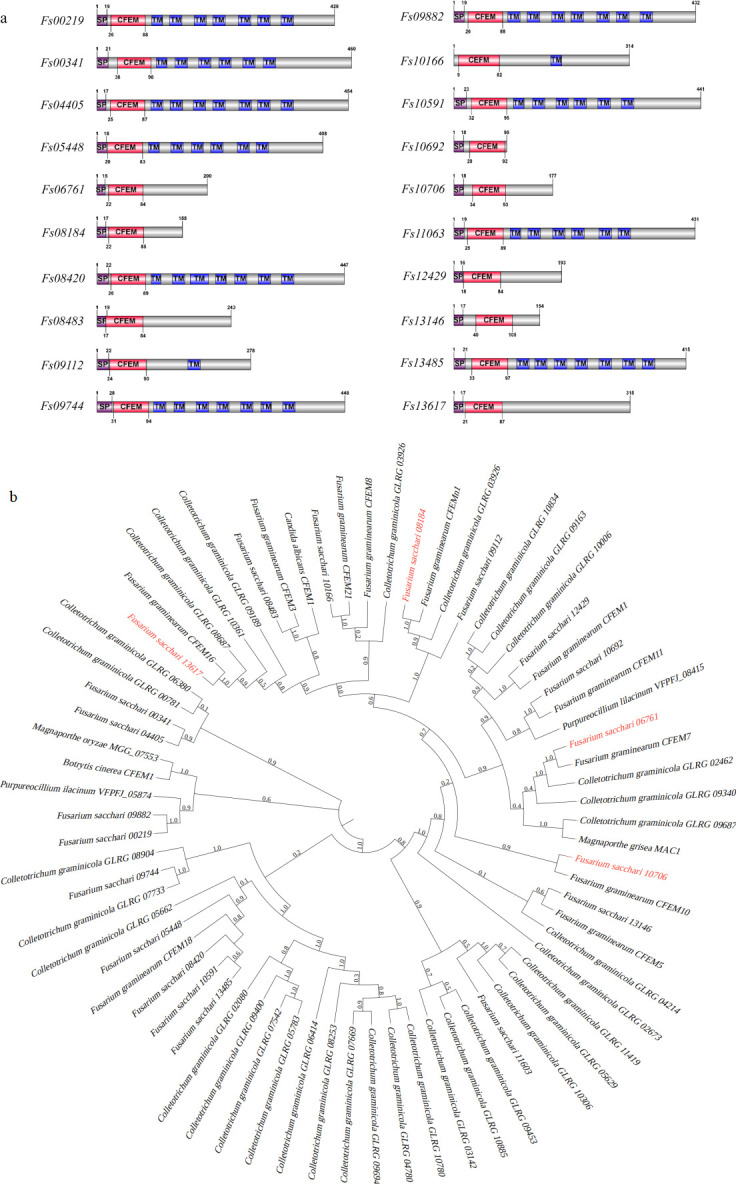
Schematic diagram of the 20 CFEM proteins and species evolutionary trees. (**a**) Amino acid sequences of 20 CFEM proteins were identified from *Fusarium sacchari*. IBS 1.0 was used to construct the schematic diagram. SP, signal peptide; CFEM, the conserved domain of the proteins; TM, transmembrane regions. (**b**) CFEM proteins reported in other pathogens were downloaded from NCBI. The phylogenetic tree was constructed following the maximum likelihood method using the FastTree software.

From the whole genome sequence of the *F. sacchari* strain, 20 proteins with CFEM domain were identified, annotated, and further analyzed using the BlastP website. Results of the analysis showed that the identified proteins range in length from 95 to 460 residues (Fig. 1a). Except for Fs10166, most FsCFEM proteins contained N-terminal signal peptides (Fig. 1a), indicating their potential secretory function. All 20 FsCFEM proteins contained 64–75 residues of the CFEM domain (Fig. 1a). Except for Fs06761, Fs08184, Fs08483, Fs10692, Fs10706, Fs12429, Fs13146, and Fs13617, the other genes had one to seven transmembrane regions. Additionally, the phylogenetic tree of 20 CFEM proteins constructed with the CFEM domains of *F. graminearum*, *M. grisea*, *C. albicans*, *B. cinerea*, and the genes of *F. graminearum* and *C. graminicola* was like the 20 FsCFEM effector proteins (Fig. 1b), suggesting that FsCFEMs were pathogenic genes.

The evolutionary tree results showed that Fs06761 was closer to FgCFEM7 and Cg02462 and that FgCFEM7 was not an important pathogenic gene, but Fs06761 had a similar function to Cg02462. Fs08184 was closer to FgCFEMn1 and Cg03926; FgCFEMn1 was an important pathogenic gene, and Fs08184 had a similar function to Cg03926. Fs13617 was a significant pathogenic gene and inhibited BAX-induced cell necrosis in this experiment, and FgCFEM16 was a non-pathogenic gene. Cg08687 did not inhibit BAX-induced cell necrosis. In conclusion, there were functional variations between the CFEM genes of different species and the FsCFEM gene, which may be due to a difference in species.

### FsCFEM effector proteins suppressed BAX-induced cell death in *N. benthamiana*

**Fig 2:**
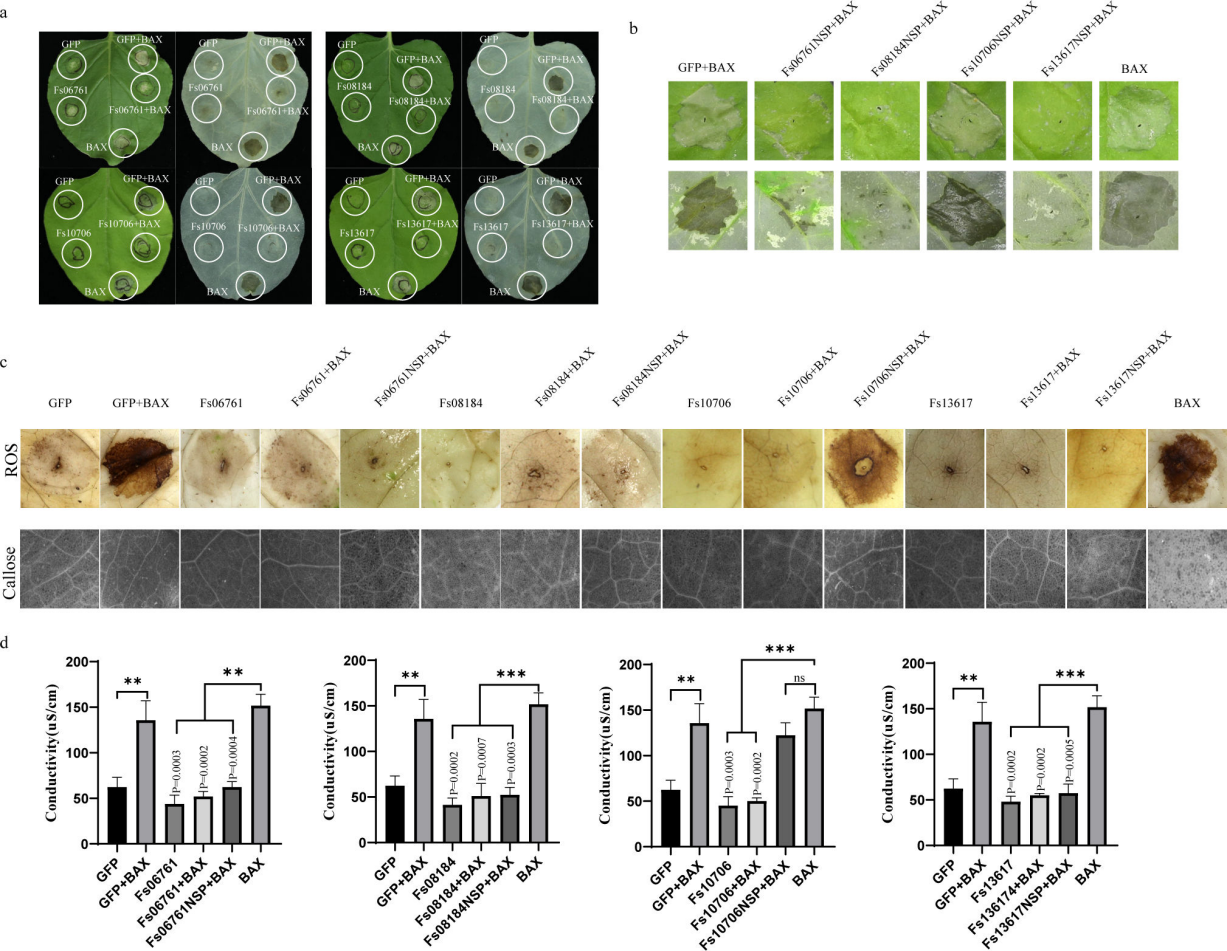
Transient expression of FsCFEM proteins in *Nicotiana benthamiana* inhibited programmed cell death triggered by BAX. (**a**) Agroinfiltration was conducted on tobacco leaves with *Agrobacterium tumefaciens* carrying the PVX: BAX gene (BAX), PVX: FsCFEM genes (CFEM), and PVX: GFP (GFP), wherein BAX and GFP were positive and negative controls, respectively. *A. tumefaciens* cells carrying PVX: BAX were inoculated after 24 h of agroinfiltration of GFP and CFEM. The results were recorded 5 days post-inoculation (dpi). To make the results more obvious, the tobacco leaves were decolorized with anhydrous alcohol. Thirty independent experiments were performed. Ten plants and 30 leaves were used for the assay. (**b**) Functional validation of FsCFEM-NSP (without signal peptide) to inhibit BAX-induced necrosis. (**c**) Validation of FsCFEMs and FsCFEMs-NSP suppressed reactive oxygen species accumulation and callose accumulation. (**d**) Induction of electrolyte accumulation by FsCFEMs and FsCFEMs-NSP.

Using transient expression experiments mediated by *A. tumefaciens* in *N. benthamiana* to explore the relationship between these CFEM proteins and hypersensitive response (HR) based on negative [green fluorescent protein (GFP)] and positive (BAX) controls, decolorization of the tobacco leaves was performed with ethanol to make necrosis more obvious. Suppression of BAX-induced cell death was observed in 4 out of the 16 cloned FsCFEM genes, namely, Fs06761, Fs08184, Fs10706, and Fs13617, which are shown (Fig. 2a). The other 12 genes showed no obvious phenotype (Fig. S1). Despite removal of the signal peptide, Fs06761-NSP, Fs08184-NSP, and Fs13617-NSP remained effective in inhibiting BAX-induced necrosis. Decolorization of the tobacco leaves with ethanol made necrosis more obvious. Reactive oxygen species spikes, callose buildup, and electrolyte leakage were significant indicators of immunological response in plants. Fs06761-NSP, Fs08181-NSP, and Fs13617-NSP retained their capacity to suppress BAX-induced cell necrosis after the signal peptide was removed, while Fs10706-NSP did not (Fig. 2b). The accumulation of reactive oxygen species and callose demonstrated the ability of inhibiting necrosis (Fig. 2c; Fig. S5). The electrolyte leakage test also demonstrated the experimental results (Fig. 2d). To assess the transcription, the injection site of each leaf was collected. The real-time PCR (RT-PCR) results confirmed the expression of the four FsCFEM genes (Fig. S2).

### Confirmation of the FsCFEM signal peptides’ secretion function

**Fig 3:**
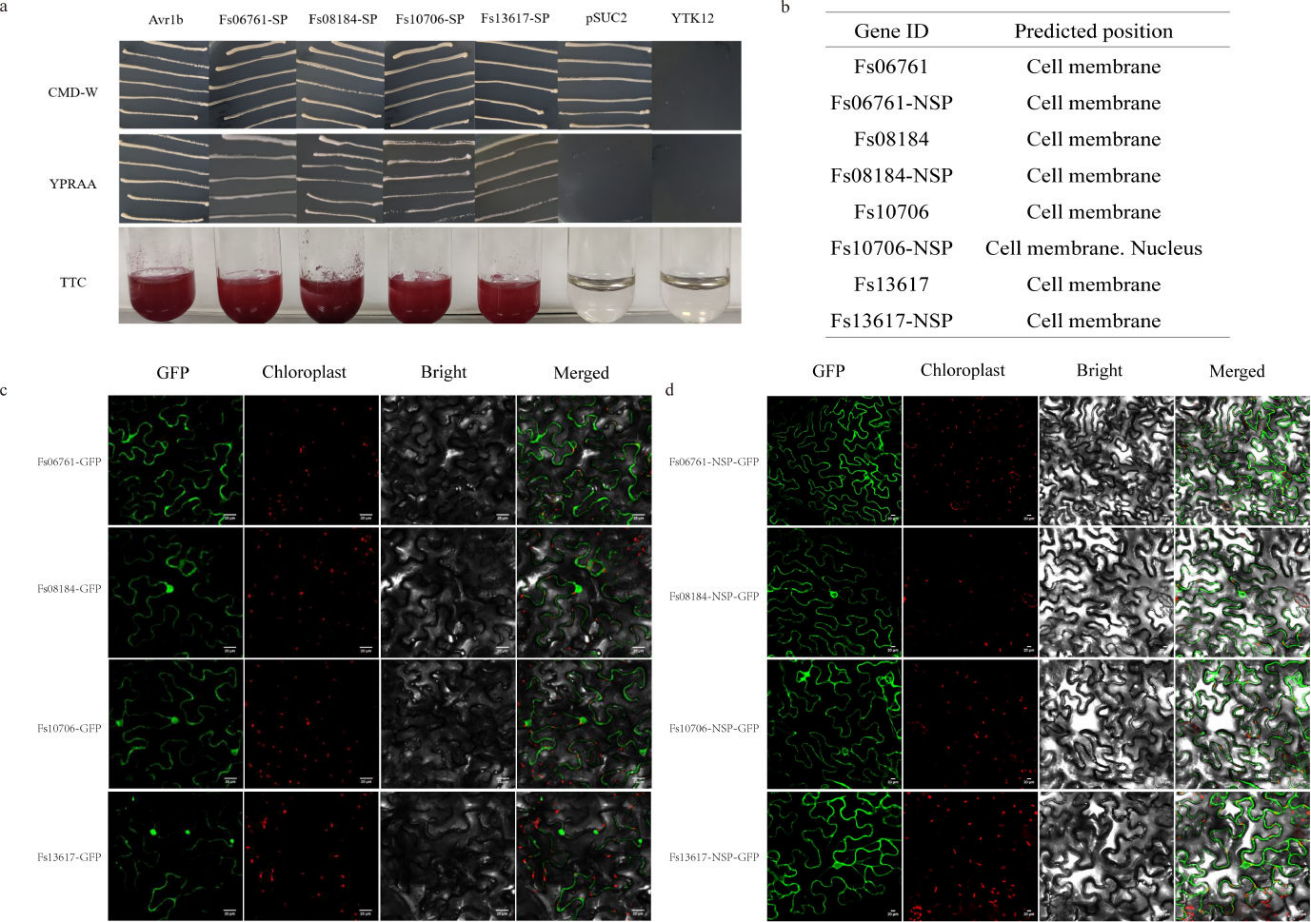
Functional validation of the putative signal peptide (SP) and localization of FsCFEMs. (**a**) Each of the recombinant plasmids of the signal peptides of the four FsCFEM proteins (Fs06761, Fs08184, Fs10706, and Fs13617) and Avr1b (positive control) were transformed into yeast with signal peptide deficiency in sucrose invertase. YTK12 transformed by recombinant vector was able to restore growth on both CMD-W and YPRAA media (1% yeast extract, 2% peptone, 2% raffinose, and 2 μg of antimycin A per liter), as well as reduce 2,3,5-triphenyltetrazolium chloride (TTC) to red 1,3,5-triphenyltetrazolium, indicating the secretion of invertase. The strain transformed by the pSUC2 plasmid (negative control) and YTK12 strain did not grow on the YPRAA medium. (**b**) Cell-PLoc 2.0 was used for predicting subcellular localization. (**c**) *Agrobacterium tumefaciens* carrying the green fluorescent protein (GFP), FsCFEMs, or the FsCFEMs-NSP fusion proteins infiltrated into 5-week-old tobacco leaves. Scale bars, 20 µm.

The yeast YTK12 secreted validation system was used to confirm the function of signal peptides (37). Signal peptides were contained in four candidate FsCFEM effector proteins at the N-terminal (Fig. 1), indicating that all four proteins were classical secreted proteins, and they were further verified through a yeast invertase secretion assay. YTK12 carrying the pSUC2 vector and Avr1b-pSUC2 was used as a negative control and positive control. In Fig. 3a, growth tests on CMD-W (0.67% yeast N base without amino acids, 0.075% tryptophan dropout supplement, 2% sucrose, 0.1% glucose) agar medium plates showed that FsCFEMs-SP and Avr1b-SP restored the secretion of invertase and resulted in yeast growth on sucrose medium. The predicted signal peptides of the four FsCFEM effector proteins restored the secretion defect of sucrose invertase of YTK12. Additionally, fructosidase SUC2 was secreted into the extracellular domain, which reduced 2,3,5-triphenyltetrazolium chloride (TTC) to red insoluble triphenyltetrazolium (Fig. 3a). These results suggest that the four effectors were typical secretory proteins.

### Subcellular localization of FsCFEM effector proteins in *N. benthamiana*

We confirmed that the four proteins can be secreted into plant cells. The four CFEM proteins were predicted to have cell membrane subcellular localization by Cell-PLoc 2.0 (Fig. 3b). FsCFEM proteins (full length and without signal peptide) were fused into the N-terminus of the GFP, and their subcellular localization was identified using transient expression analysis in *N. benthamiana*. Fig. 3c and d show the location of the proteins in the plant cells. The precise locations of the four full-length proteins were determined through co-localization, with DAPI used as the nuclear localization signal and OsMCA1 used as the cell membrane marker (Fig. S3). These four full-length proteins were secreted into plant cells based on their co-localization results. The subcellular localization assay based on fusion with GFP supported that effector proteins were transported into plant cells to perform their functions.

### Expression pattern analysis of FsCFEM effector proteins in the *Fusarium*-infected sugarcane

**Fig 4:**
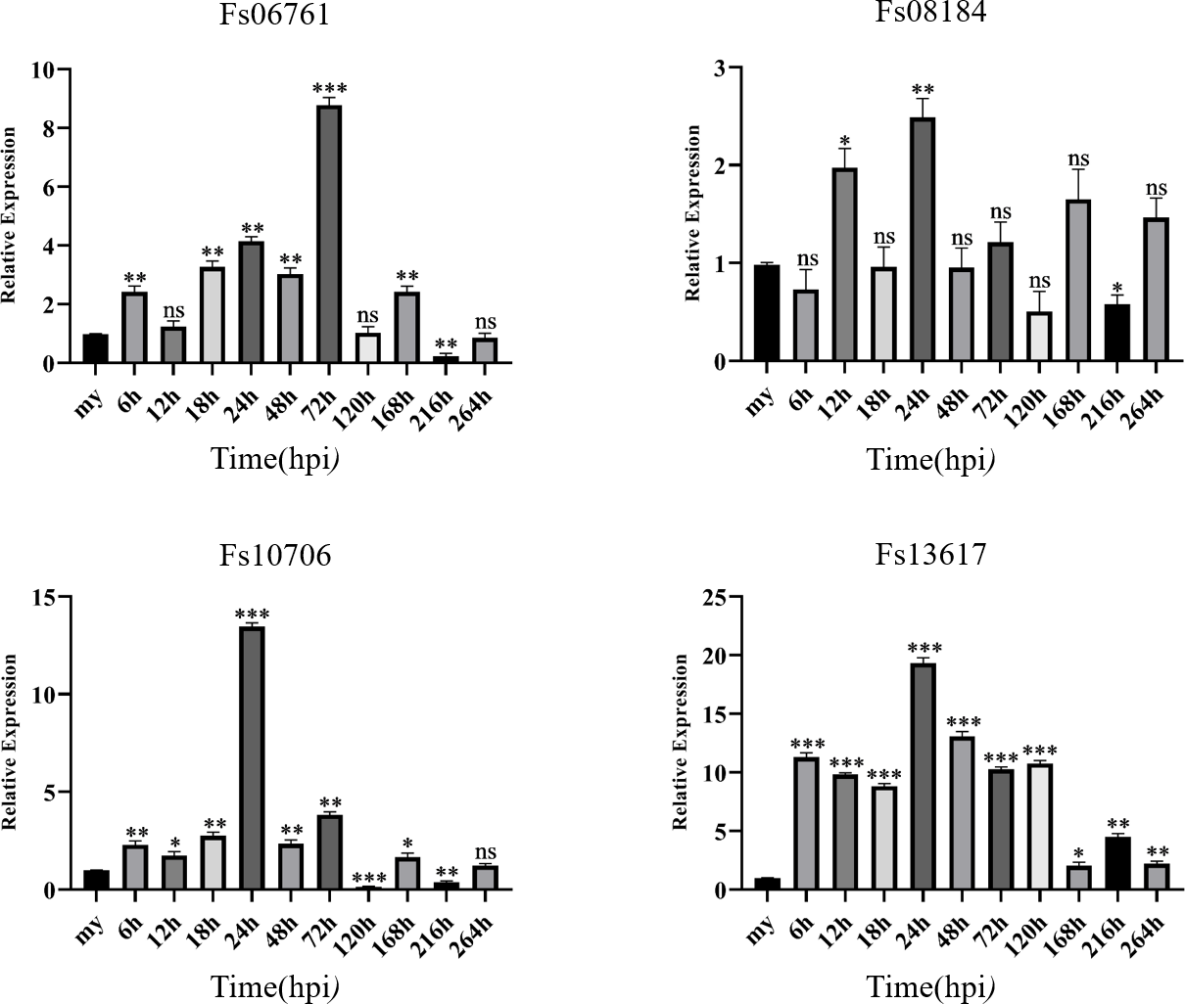
Gene expression patterns of four CFEM proteins in sugarcane plants detected with qRT-PCR after inoculation. Sugarcane leaves inoculated with *F. sacchari* were sampled at 6, 12, 18, 24, 48, 72, 120, 168, and 264 h post-inoculation. The expression levels of each gene were calculated employing the 2−ΔΔCt method using *Fusarium sacchari* actin as the reference gene. The expression level of FsCFEM in mycelia of *F. sacchari* before inoculation of sugarcane plants at mycelia (my) was defined as baseline. Values represent the means and standard deviations of three independent replicates (ns, no significant difference; *P < 0.05; **P < 0.01; ***P < 0.001; statistical significance was determined using two-way analysis of variance).

To explore the possible functions of the four genes during the infection process between *F. sacchari* and sugarcane, we investigated the expression patterns of these four genes at different infection stages after *F. sacchari* conidia were inoculated in sugarcane. Figure 4 shows the quantitative real-time PCR (qRT-PCR) expression pattern of four FsCFEM proteins. Expression levels of all four effector genes increased at first and then decreased, and some exhibited the highest expression levels at 24 h post-inoculation (hpi). The expression levels of these four genes increased during their interaction with the host sugarcane, and the highest expression at 24 hpi indicated that these four genes might play an essential role at 24 hpi. Following a previous study (38), at 24 hpi, the fungal spores appeared swollen, and the mycelia had spread to the trichomes at 72 hpi. Furthermore, the expression level of Fs06761 suddenly and significantly increased to the highest level at 72 hpi, suggesting that it may be related to infection and colonization. The expression level of Fs08184, Fs10706, and Fs13617 peaked at 24 hpi, suggesting that they may be involved in the germination of fungal spores. Compared with the expression levels of Fs08184 and Fs10706, the function of Fs13617 may be more important than that of the others and has great significance when fungal spores are swollen. These results strongly suggest that the four genes play an important role during the infection process.

### Fs06761, Fs08184, and Fs13617 are virulence factors needed for the full virulence of *F. sacchari*

**Fig 5:**
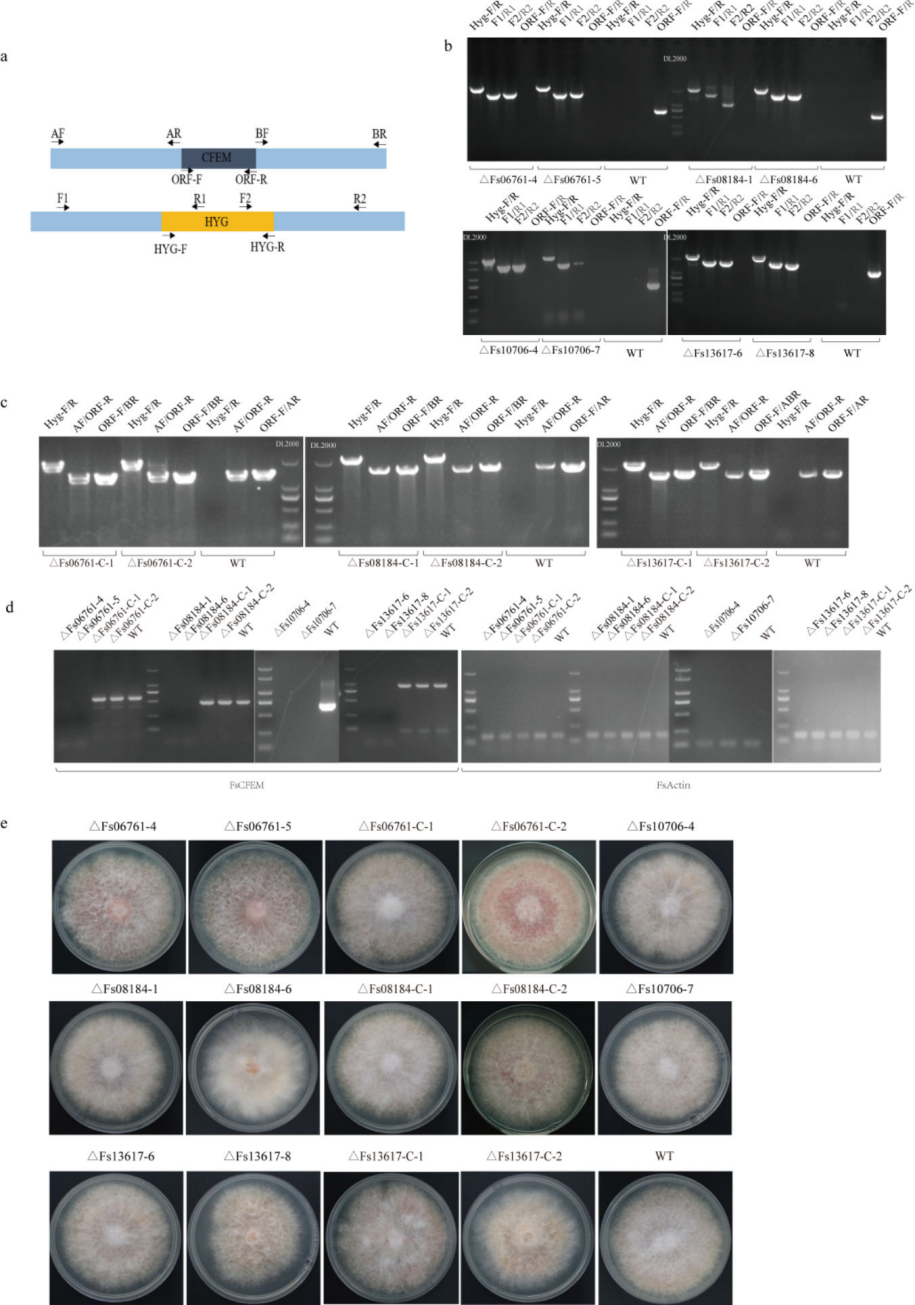
FsCFEM knockout and pathogenicity tests. (**a**) The model of gene deletion strategy for FsCFEMs. (**b**) Agarose gel electrophoresis of PCR products to identify the mutants. Lane Hyg-F/R, primers Hyg-F/Hyg-R for hygromycin resistance gene; lane F1/R1, FsCFEMs-P800F/Hyg800R; lane F2/R2, Hyg1300F/ FsCFEMs-P800R. ∆Fs06761-4, ∆Fs06761-5, ∆Fs08184-1, ∆Fs08184-6, ∆Fs10706-7, ∆Fs13617-6, and ∆Fs13617-8 are candidates: wild-type (WT) strain *F. sacchari*. (**C**) Agarose gel electrophoresis of PCR products to identify the complemented mutants. (**d**) RT-PCR to identify the knockout mutants and complemented mutants. (**e**) Colony morphology of WT knockout mutant and complemented mutant strains on potato dextrose agar for 7 days at 28°C.

**Fig 6:**
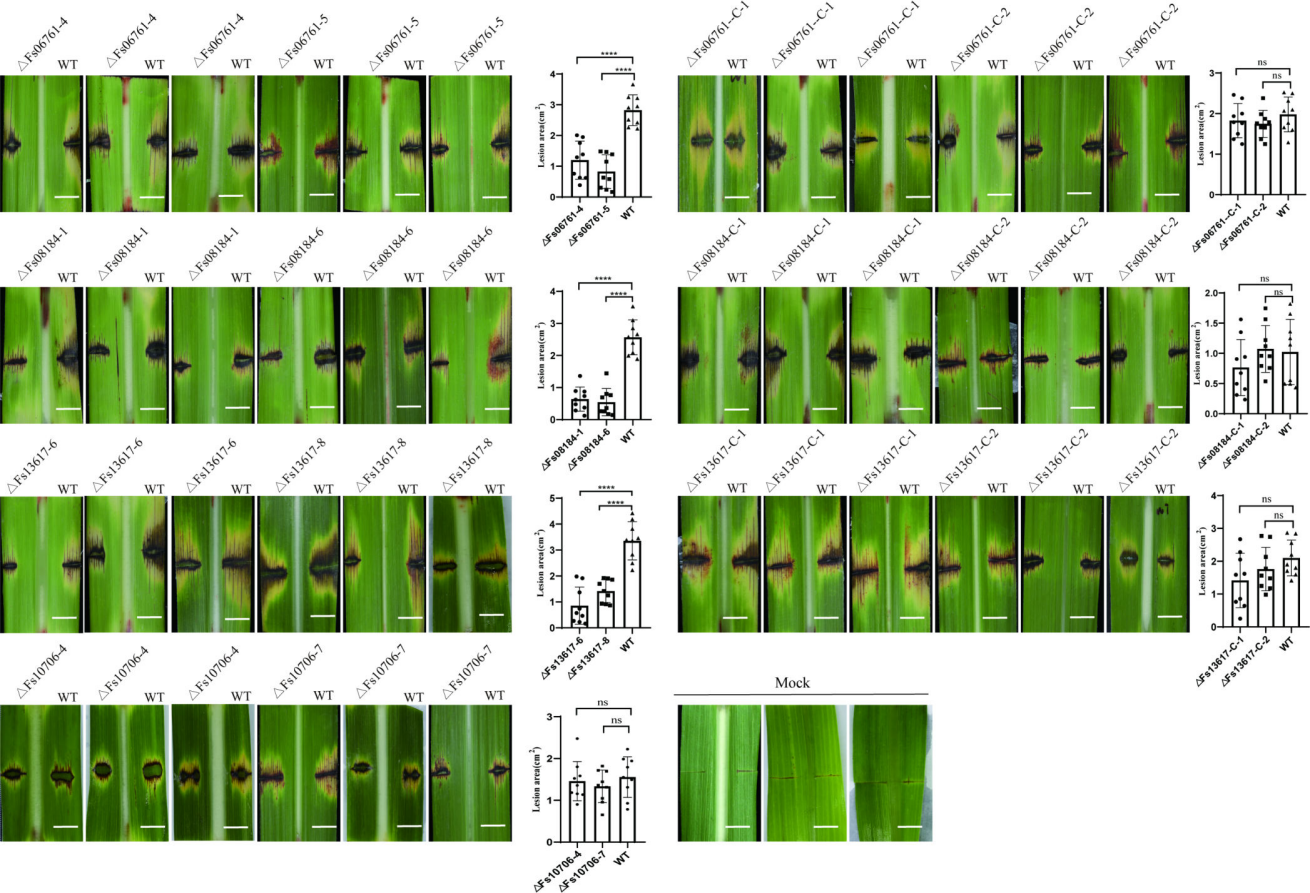
Detached leaves from “Zhongzhe 1” were inoculated with a fungal plaque of the wild type (WT), deletion mutant FsCFEMs, or double deionized water as a mock control. The leaves were kept under darkness for 3 days and then photographed. ImageJ (developer info) was used to calculate the lesion area (39). Asterisks indicate significant differences in gene expression levels relative to the WT: ns, no significant difference; *P < 0.1; **P < 0.05; ***P < 0.01; ****P < 0.001. Statistical significance was determined using two-way analysis of variance. Bar, 1 cm. Each assay was repeated on at least nine leaves, and the other results showed in Fig. S4.

To identify the biological function of FsCFEMs, we knocked out FsCFEMs in wild-type (WT) strains using a split-tagging approach (Fig. 5a). The FsCFEM deletion mutants ∆Fs06761-4, ∆Fs06761-5, ∆Fs08184-1, ∆Fs08184-6, ∆Fs10706-7, ∆Fs13617-6, and ∆Fs13617-8 had the FsCFEM gene successfully replaced with the hygromycin gene (Fig. 5b). To produce supplemented mutant strains, complement assays were performed using the marker gene G418; the complemented mutant strains were then verified by polymerase chain reaction (PCR) and RT-PCR (Fig. 5c and d). However, there was no significant change in phenotype between the mutant and wild type (Fig. 5e). To evaluate the role of ∆FsCFEMs in pathogenesis, an *in vitro* inoculation experiment was carried out on sugarcane leaves. The pathogenicity test showed that virulence to pokkah boeng disease of ∆FsCFEMs was significantly weakened, and the necrotic area of the wild type was larger than that of the mutant (Fig. 6; Fig. S4). Additionally, gene deletion of the four FsCFEMs reduced the pathogenicity. Among them, ∆Fs06761-4, ∆Fs08184-6, and ∆Fs13617-1 were more closely associated with the pathogenicity than ∆Fs10706-7. Compared with ∆Fs06761-4 and ∆Fs08184-6, the necrotic area of ∆Fs13617-1 decreased more than that of the wild type. Therefore, the FsCFEMs gene encodes core virulence factors for *F. sacchari*.

## DISCUSSION

In general, the successful development of disease and the determination of host–pathogen compatibility require secreted proteins. The effector proteins secreted by pathogenic bacteria can act on the inside and outside of plant cells, interact with a variety of targets, block PAMP-triggered immunity (PTI) or effector-triggered immunity, reduce plant immunity, and help pathogenic bacteria survive in host or non-host plants (40). In fungal–plant interactions, fungi often secrete effectors that interfere with host defense mechanisms and generate biotrophic infection structures to absorb host cell nutrients (41, 42). PBD, the causal agent of *F. sacchari*, is a serious disease of sugarcane that causes substantial reduction in yield and quality. Knowledge of its pathogenic mechanism could provide important insights for disease resistance breeding in sugarcane. To date, reports about effector proteins from *F. sacchari* are limited. Our study identified that four FsCFEM proteins play a role in the infection stage and suppressing plant immunity, which provides insights for the molecular mechanisms of *F. sacchari*–sugarcane interaction.

The CFEM protein family is a vital virulence factor in fungi, and annotated CFEM effector functions are important in pathogen–host interactions. The number of CFEM proteins in various fungi has been identified, and pathogenesis has been examined (18, 25). The results showed that CFEM domain proteins are present in *F. graminearum* (22, 23) and *M. grisea* (19), and there are six CFEM domain proteins in *C. albicans* (26), 24 CFEM proteins in *C. graminicola* (24), and three CFEM proteins in *B. cinerea* (31). In our study, 20 CFEM proteins were identified in *F. sacchari* from its genomic sequence. Based on the evolutionary trees, the majority of the FsCFEM proteins were close homologs with *F. graminearum* and *C. graminicola*. Despite functional differences between FsCFEM and FgCFEM proximate genes, which may be attributed to species differences, Fs06761, Fs08184, and Fs13617 remained important pathogenic factors for *F. sacchari*. These three genes will be a breakthrough for further exploring the relationship between FsCFEMs and plant immunity.

CFEM effector proteins act at different sites in a plant cell, but in general, they operate in the cell membrane (11). In a subcellular localization study of five CgCFEM effector proteins of *C. graminicola*, CgCFEM6 and CgCFEM8 were found to accumulate in the nucleus, CgCFEM7 and CgCFEM9 were located in the cell membrane, and CgCFEM15 was expressed in nucleus and cell membrane of *N. benthamiana* (24). According to our research, the host cell’s ability to release FsCFEMs increases the structural and functional diversity of the cell. Moreover, secretion and subcellular experiments revealed that CfEC92 was transported into the host cells by fungal cells and then secreted from them to affect the host (43). Subcellular localization analysis in our investigations showed that four FsCFEM proteins were predominantly localized to the cell membrane and nucleus. Similar with the localization of other CFEM proteins, the location of the cell membrane could be linked to the GPI domain and could enter the cell via this binding and subsequently function within the cell.

Host receptors can recognize phytopathogenic PAMPs or effectors to elicit strong host cell defense, such as HR. The BAX protein belongs to the death-promoting Bcl-2 family and has been shown to induce cell death (such as defense-related HR) when expressed in tobacco leaves (44). Therefore, BAX-induced cell death has been regarded as a valuable reference for selecting effector proteins (45). Thus far, many pathogen effectors have been identified through the *Agrobacterium*-mediated transient expression system in tobacco (46 – 48). PstGSRE1 in *Puccinia striiformis*, CfEC92 in *Colletotrichum fructicola*, and five CFEM effectors in *C. graminicola* were found to suppress BAX-induced cell death (24, 43, 49). In *L. theobromae* and *V. dahlia*, the effector proteins were found to induce cell death in tobacco leaves (27, 50). Therefore, we speculated that these FsCFEM genes may act as effector proteins to disturb the plant immunity response. Although the function of FsCFEM proteins is different with *L. theobromae*, it may due to genetic differences between different pathogens. The function of CFEM gene was different from diverse strains. In the process of cross-evolution between some strains and host plants, the CFEM gene was recognized by pattern recognition receptor evolved by host plants. Resulting in LtCFEMs proteins could induce plant immune response. However, such speculation needs to be verified by experiments using yeast two-hybrid. Interestingly, Fs10706 lost its ability to inhibit necrosis after removing the signal peptide, suggesting that the signal peptide is more important for Fs10706. However, without the signal peptide, the subcellular location of the Fs10706 protein remained in the plant cell. The signal peptide would be cleaved off after effector proteins enter the plant cell. In general, the loss of the signal peptide does not affect the function of the protein. It is speculated that Fs10706 lost the ability during evolution but may be given the other functions that need to be investigated.

In a previous study, the secreted proteins were confirmed to affect the pathogenicity of the pathogen during the infection stage (51). During the infection stage, the transcript levels of BcCFEM1 in *B. cinerea* increased from 12 to 36 hpi and then maintained higher expression than values at mycelia (30). The fungal spores of *F. sacchari* are swollen at 24 hpi, and mycelia spread to the trichomes at 72 hpi (38). In our study, Fs06761 may be related to infection and colonization. Fs08184, Fs10706, and Fs13617 may be involved in the germination of fungal spores. The four CFEM proteins were involved in the process of plant infection, even though they were not correlated with the growth and development of pathogen. In the future, we will monitor the changes in these genes during infection by constructing fluorescent strains. Therefore, we speculated that these four proteins have disparate roles in the pathogen infection process of sugarcane.

In this study, the CFEM-containing proteins displayed differentiation in their virulence function on sugarcane. Compared to the WT, ∆FsCFEMs showed no difference in colony morphology on potato dextrose agar (PDA) plates. In *Botrytis cinerea*, the deletion of BcCFEM1 does not affect the growth rate, conidial germination, and colony morphology (30). The virulence of ∆Fs06761, ∆Fs08184, and ∆Fs13617 was weakened. Similar roles that contribute to virulence have been demonstrated for the CFEMs in *Fusarium graminearum* (23) and *Fusarium oxysporum* species complex (Fosc) (52). FoSIX6 knockouts of *F. oxysporum* f. sp. show that it was closely related to virulence (53). Virulence analysis found that Fs06761, Fs08184, and Fs13617 were closely related to virulence. Fs10706 was not closely related to virulence, perhaps due to pathogen evolution, which may involve pathogen development or stress resistance. The functional diversity of CFEM genes in pathogen growth and infection will be further explored. The reduced pathogenicity of ∆FsCFEMs with pleotropic defects indicates that FsCFEMs are virulence factors.

### Conclusion

In summary, we identified four CFEM proteins from *F. sacchari* that suppress BAX-triggered PCD in *N. benthamiana*. The four CFEM proteins functioned at the early infection stage of *F. sacchari* on sugarcane. The molecular mechanisms by how CFEM proteins suppress host defenses remain unclear. Pathogenic effectors have been identified to target multiple host plant proteins, resulting in suppression of the host immune response (54, 55). Since CFEM proteins function in host cells, our future studies will focus on identifying possible targets of CFEM proteins in sugarcane to elucidate the molecular basis of plant defense inhibition.

## MATERIALS AND METHODS

### Plants, microbes, and growth conditions

The *F. sacchari* (SRR18652388) was isolated from diseased sugarcane leaves. The strain routinely grows on PDA (Solarbio, Beijing, China) media. The *Agrobacterium tumefaciens* strain GV3101 (with pJIC_SARep) was used for *Agrobacterium*-mediated transient gene expression in tobacco (*N. benthamiana*) plant leaves. *Escherichia coli* strain Top10 was used to propagate plasmids. The yeast (*Saccharomyces cerevisiae*) mutant YTK12 was used to verify the secretory function of signal peptides. Sugarcane cultivar “Zhongzhe 1” and tobacco (*N. benthamiana*) were grown with a photoperiod set at 16:8 h (day:night) and temperature set at 28°C:25°C and 25°C:22°C for sugarcane and tobacco, respectively.

### Construction of plasmids

To clone the FsCFEM genes, specific primers were designed using the NCBI primer design tool (https://www.ncbi.nlm.nih.gov/tools/primer-blast/). Supplementary Table S1 lists the primers used for plasmid construction. Total RNA was isolated using a Quick RNA isolation kit (TIANGEN, China). The RNA was reversely transcribed into cDNA using a 1st Strand cDNA Synthesis Kit (Vazyme, China). The PVX vector was used in agroinfiltration for transient gene expression in *N. benthamiana* plants. A 2× Phanta Mix (Vazyme, China) was used to clone genes by PCR. Plasmids pSUC2 and pBWA(V)HS were used to confirm the secretion function and subcellular localization of the FsCFEM proteins. The ORF of FsCFEMs fused with the GFP at N-terminal without termination codon.

### *In silico* analysis of CFEM proteins in *F. sacchari*

FsCFEM proteins were screened from the whole genome sequence of the *F. sacchari* strain based on data available at the BlastP annotations (https://blast.ncbi.nlm.nih.gov/Blast.cgi?PROGRAM=blastp&PAGETYPE=BlastSearch&LINKLOC=blasthome). The CFEM domain and transmembrane regions were obtained through bioinformatics analysis by SMART website (http://smart.embl-heidelberg.d). Additionally, the signal peptides of these proteins were analyzed using the bioinformatics tool, SignalP-5.0 Server (http://www.cbs.dtu.dk/services/SignalP/). To predict the subcellular localization of the four genes, Cell-PLoc 2.0 (http://www.csbio.sjtu.edu.cn/bioinf/Cell-PLoc-2/) was used. All the sequences were obtained from the NCBI website (https://www.ncbi.nlm.nih.gov/), and multiple sequence alignment was completed in the MAFFT website using the default parameters (56) (https://mafft.cbrc.jp/alignment/server/index.html). The maximum likelihood method was used to construct the phylogenetic tree using the FastTree software’s preset model (JTT + CAT model) (57, 58).

### *Agrobacterium*-mediated transient expression in *N. benthamiana*

The HR of FsCFEM proteins was assayed on *N. benthamiana* leaves to determine the purpose of these genes in PCD. BAX was used as a positive control in the agroinfiltration assay (43). GFP-PVX was used as a negative control. The whole ORFs of FsCFEM genes were constructed into transient expression vector PVX. *A. tumefaciens* GV3101 containing the recombinant plasmid were used for the HR assay (59). *A. tumefaciens* was grown in Luria-Bertani broth containing corresponding antibiotics (50 µg/mL of kanamycin, 40 µg/mL of gentamicin, 20 µg/mL of rifampicin) in 220 rpm for 24 h at 28°C. The bacteria were centrifuged at 5,000 rpm for 5 min, and the bacteria pellets were re-suspended three times with 10 mM MgCl_2_ (OD_600_ = 0.4). Specifically, agrobacteria suspensions were placed in the dark at 28°C for 2 h before infection. The agroinfiltration assay was carried out on the bottom leaf surface of 1-month-old tobacco plants. In addition, the BAX was inoculated after 24 h in the same spot of the tobacco leaves where FsCFEM genes were inoculated earlier, and the effects were photo documented for 5 days. At 3 days post-inoculation (dpi), the treated leaves were collected and observed under a Leica TCS SPE confocal laser-scanning microscope at 488-nm excitation; 512-nm (GFP) and 656-nm (chloroplast auto-fluorescence) emissions were used for subcellular localization analysis. The tobacco leaves were collected at 4–5 dpi when BAX-induced PCD became apparent with alcohol decolorization. Each assay was repeated on at least three leaves on three different plants.

### DAB staining

The reactive oxygen species (ROS) burst in plant tissue was examined by staining with diaminobenzidine (DAB) as described previously. Leaf pieces were infiltrated with DAB solution (Sigma) (1 mg/mL, pH 3.8) and shaken at room temperature for 12  h in the light. The leaf pieces were then destained with 95% ethanol, and photographs were taken.

### Trypan blue staining

*Nicotiana benthamiana* leaves expressing recombinant vectors or empty vectors were stained by boiling for 10 min in lactophenol–trypan blue solution (10-mL lactic acid, 10-mL glycerol, 10-g phenol, 10-mg trypan blue, 10-mL distilled water). They were then decolored with gentle shaking in a chloral hydrate solution (2.5 g/mL) for 12  h. Samples were photographed under natural light.

### Electrolyte leakage

Cell death was quantified by determining electrolyte leakage using a previously described method. Samples from *N. benthamiana* (diameter, 1 cm) were immersed in Nanopure water (1 mL) for 3 h at room temperature to determine the electrical conductivity. A conductivity meter (SX-650, Sanxin, Shanghai, China) was used to measure the conductivity. This assay was repeated three times.

### Yeast secretion assay

Following the previous study, the yeast YTK12 system was used to identify the secretion function of the predicted signal peptides of the four CFEMs (60). The pSUC2 vector contained the tryptophan synthesis-related gene but lacked the signal peptide and the invertase gene without the start codon. Therefore, only inserting the secreted gene activated the SUC2 gene and was secreted into the medium, converting sucrose to glucose, which is required for yeast growth. YTK12 transformed with pSUC2-Avr1bSP and the empty vector pSUC2 were used as positive and negative controls (49). The transformation method was described in a previous study (38). Yeast carrying pSUC2 was grown on CMD-W (lacking tryptophan) media. If the signal peptide had the secretion function, the clones grew on YPRAA media (1% yeast extract, 2% peptone, 2% raffinose, and 2 μg of antimycin A per liter).

### Expression pattern analysis of FsCFEM effector proteins

The *F. sacchari* conidial suspensions (5 × 10^5^ conidia/mL) were injected into the apical meristem leaf of 1-month-old sugarcane plants. Total RNA was isolated from the inoculated leaves collected from mycelia at 6, 12, 18, 24, 48, 72, 120, 168, 216, and 264 h. Every treatment had three independent replicates, using mycelia as the control group. Primer Premier 5 was used to design the qRT-PCR primers (Table S1) based on each full-length FsCFEM gene sequence. qRT-PCR was performed using a LightCycler 96 system (Roche, Switzerland) and TB Green (Takara Biomedical Technology, Beijing, China). *Fusarium sacchari* actin was used as a reference gene following the relative expression method (61). Relative expression levels were determined from three independent biological replicates using the 2^−ΔΔCt^ method (38).

### Transformation of *F. sacchari*

For *F. sacchari* transformation, polyethylene glycol-mediated fungal genetic transformation was similar to that for *Fusarium pseudograminearum* (62). We constructed a homologous recombination fragment containing flanking sequences of the target gene and hygromycin resistance gene (Hph). The 5′- and 3′-flanking regions (approximately 1,000 bp each) of the target gene were amplified via primer pairs FsCFEMs-AF/AR and BF/BR. The target fragment (about 4,600 bp) was constructed by double-joint PCR. Putative transformants were selected on PDA medium containing 50 µg/mL hygromycin, with three replicated plates per strain. Inoculation of sugarcane leaves was used to verify the pathogenicity. Each inoculation experiment had at least three repeats.

### Statistical analysis

All experiments involving measurements, quantifications, and imaging were repeated at least three times with similar results. GraphPad Prism version 8 was used for statistical tests. In all graphs, asterisks mark statistical significance (*P < 0.05; **P < 0.01; ***P < 0.001; ns, not significant) according to two-way analysis of variance (Student’s *t*-test).

## Data Availability

The strain *Fusarium sacchari* (SRR18652388) was uploaded on the NCBI database. The nucleotide sequences of TEF-1α, RBP1, and RBP2 were detected to identify the strain. The genomic DNA library was generated using an Illumina HiSeq 2500 by commercial sequencing.

## SUPPLEMENTAL MATERIAL

Supplemental material spectrum.01452-23-s0001.docx Fig. S1 to S5; Tables S1 to S3.

ASM does not own the copyrights to Supplemental Material that may be linked to, or accessed through, an article. The authors have granted ASM a non-exclusive, world-wide license to publish the Supplemental Material files. Please contact the corresponding author directly for reuse.
