# Supplementary material for: Identification of common fungal extracellular membrane (CFEM) proteins in Fusarium sacchari that inhibit plant immunity and contribute to virulence
Source: Microbiol Spectr. 2023 Nov 14;11(6):e01452-23. doi: 10.1128/spectrum.01452-23 (PMC10715082; doi:10.1128/spectrum.01452-23)
Supplement: Supplemental material — Fig. S1 to S5; Tables S1 to S3. [file spectrum.01452-23-s0001.docx]

Supplementary Materials:

Figure S1. Of the 20 CFEM genes, only 16 genes were successfully cloned,

Figure S2. RT-PCR was used to confirm gene transcription in tobacco leaf tissue.

Figure S3. Co-Localized was used to obtain the precise location of the four proteins.

Figure S4. Using in vivo inoculation, the pathogenicity of mutants is discovered.

Figure S5. Callose deposition was determined using a fluorescence microscope using a UV filter after stained with aniline blue, and the spotlights were counted by ImageJ.

Table S1. List of primers used for plasmid construction of FsCFEM genes

Table S2. The nucleotide sequence of TEF-1α, RBP1 and RBP2

Table S3. The nucleotide sequence of 20 CFEM genes


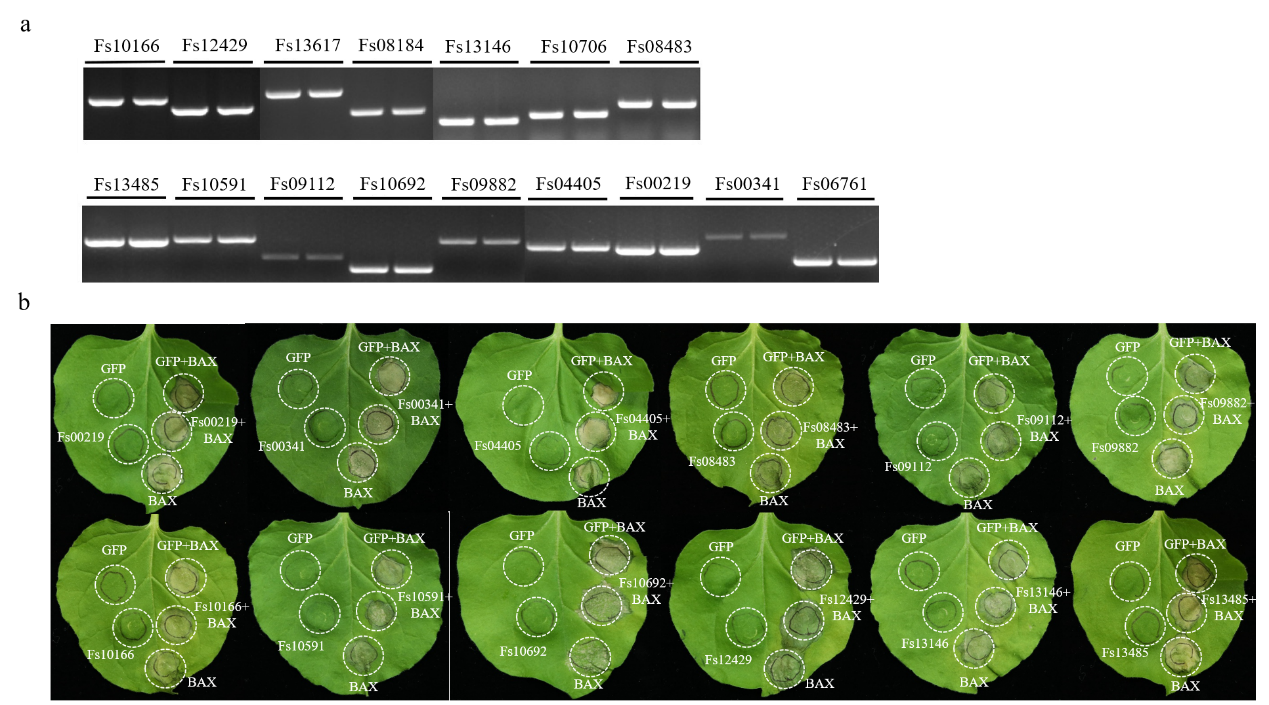


Figure S1. Of the 20 CFEM genes, only 16 genes were successfully cloned, while the other 12 CFEM proteins failed to induce or suppress cell death in *N. benthamiana*. (a) Of the 20 CFEM genes, only 16 genes were successfully cloned. (b) 12 showed a non-obvious phenotype on tobacco leaves by *Agrobacterium*-mediated transient expression.


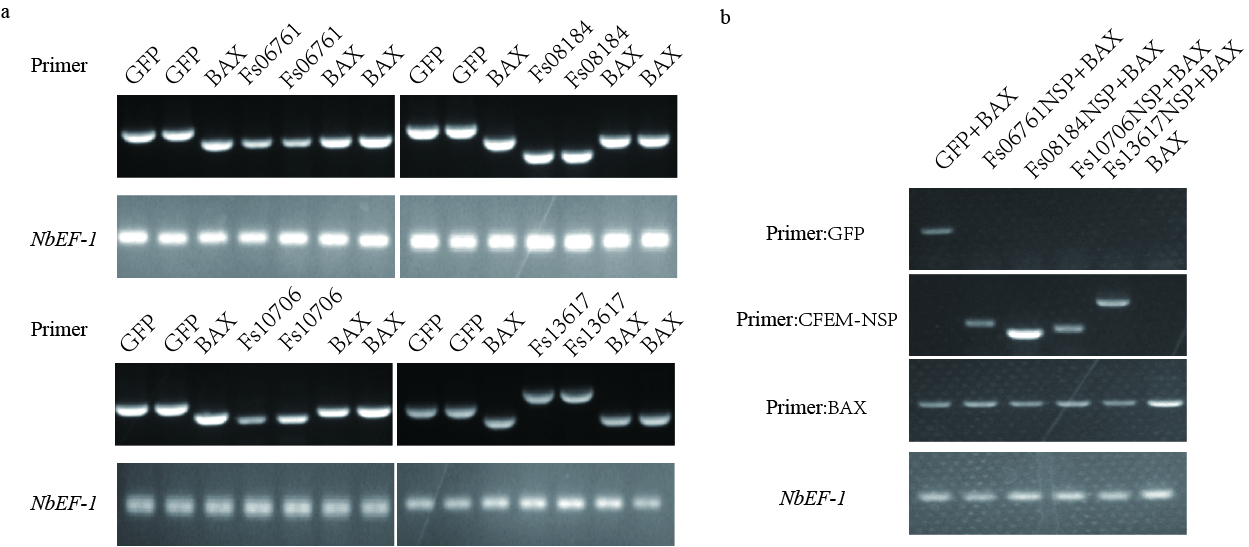


Figure S2. RT-PCR was used to confirm gene transcription in tobacco leaf tissue.


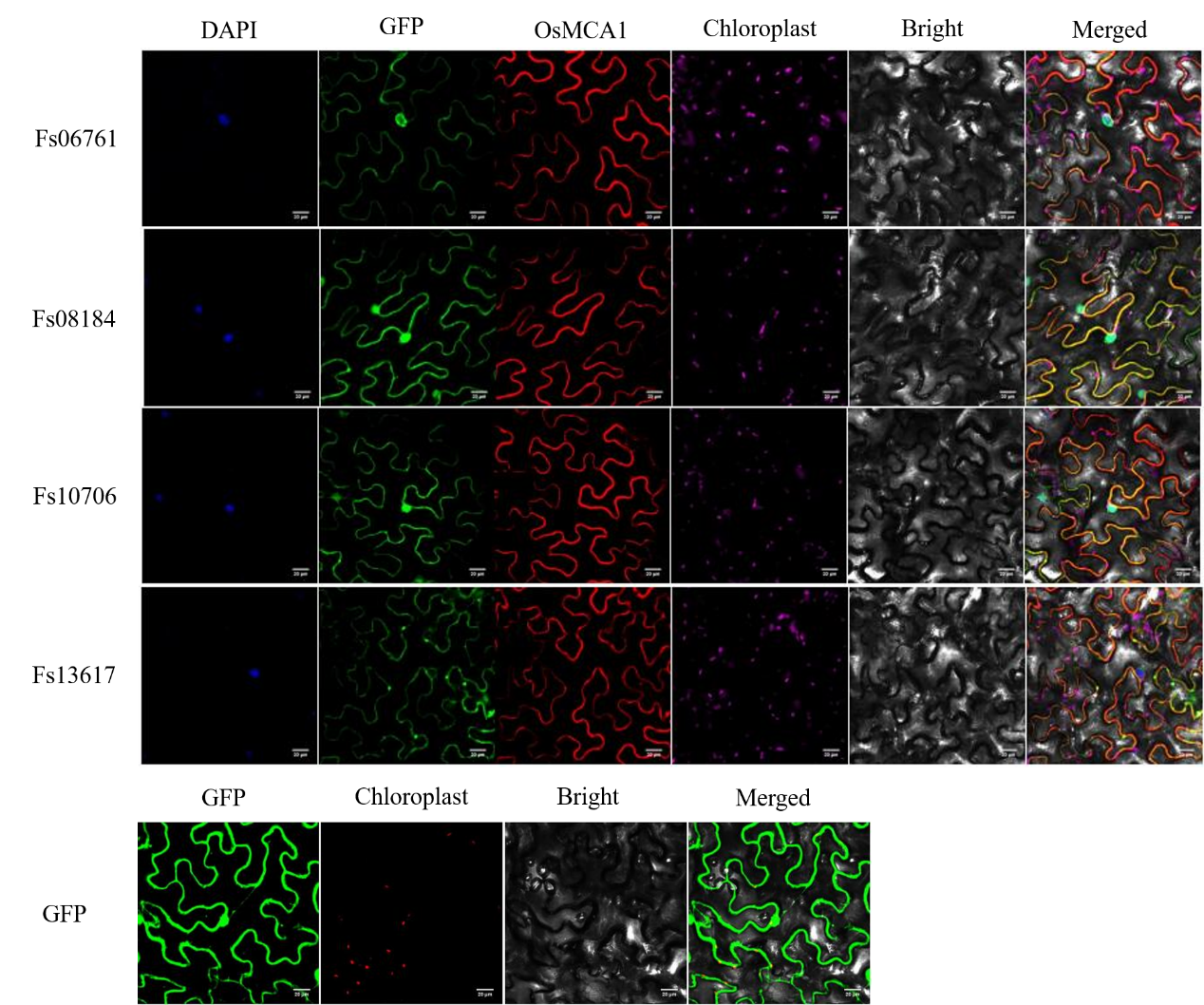


Figure S3. Co-Localized was used to obtain the precise location of the four proteins. A nuclear localization signal was used by DAPI, and OsMCA1 was used as the cell membrane marker. Scale bars = 20 µm.


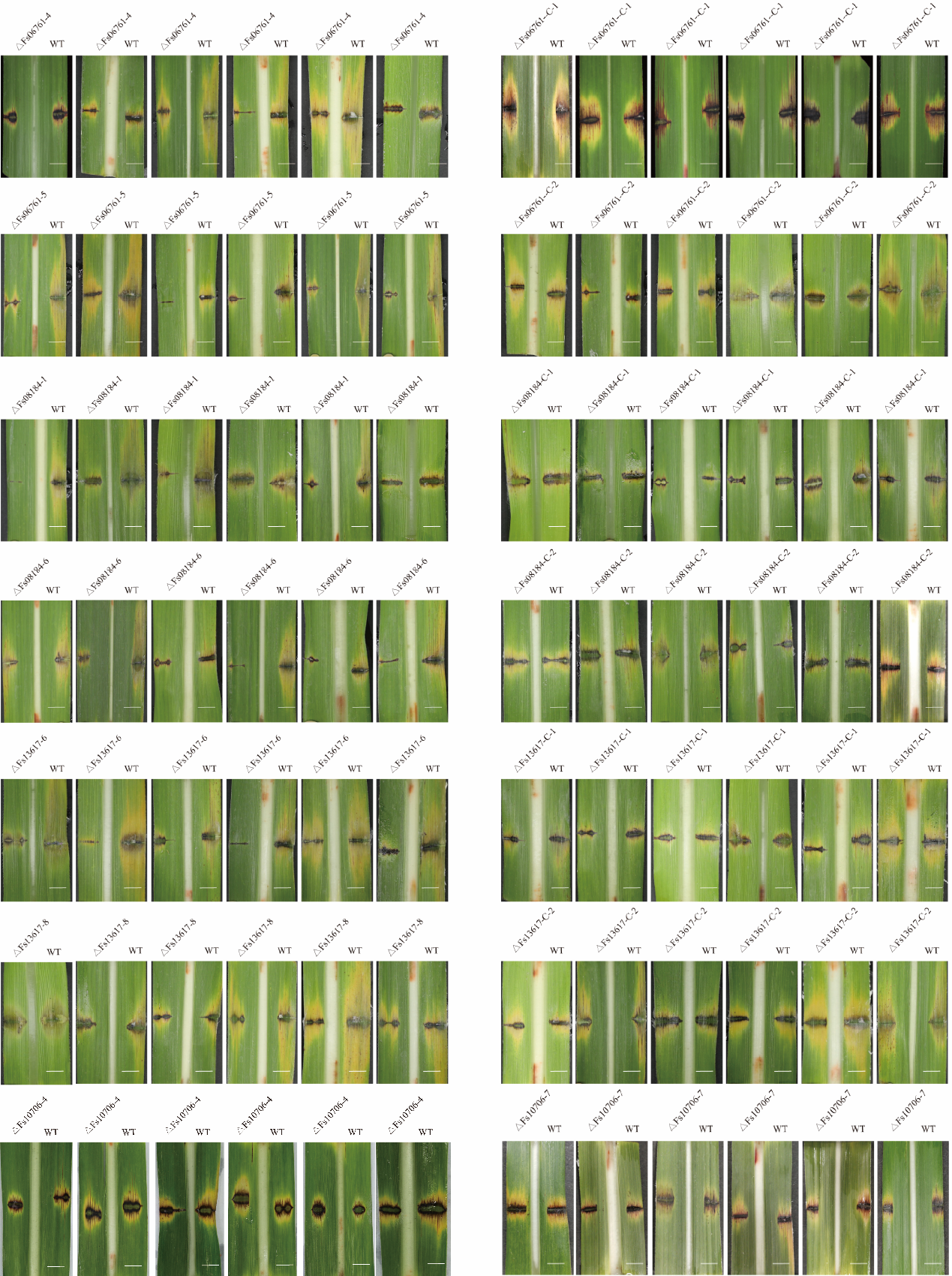


Figure S4. Using in vivo inoculation, the pathogenicity of mutants is discovered.


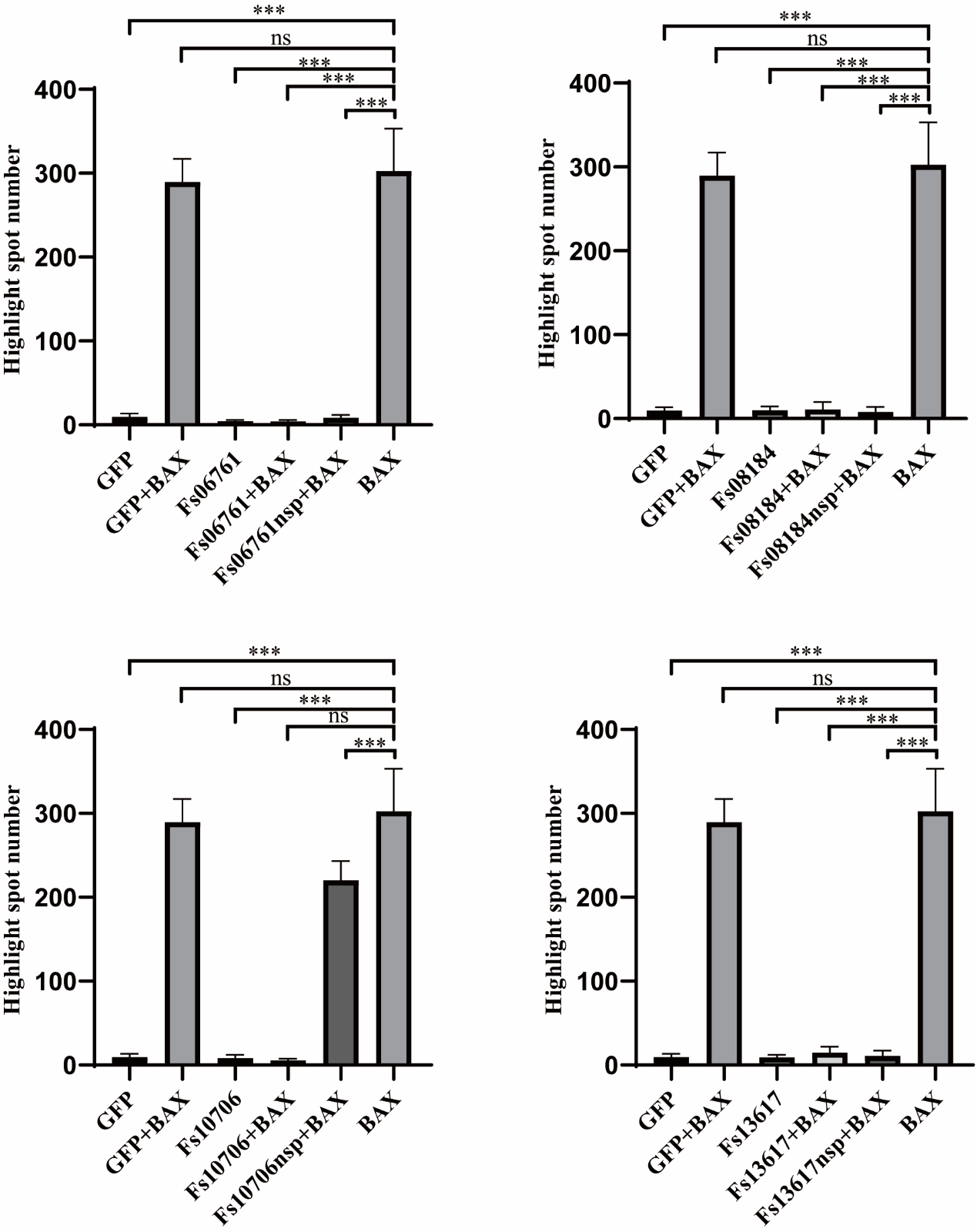


Figure S5. Callose deposition was determined using a fluorescence microscope using a UV filter after stained with aniline blue, and the spotlights were counted by ImageJ.

**Table S1. List of primers used for plasmid construction of FsCFEM genes**

| **Primer name** | Sequence (5'-3') | **Purpose** |
| --- | --- | --- |
| Fs00219-Not I F | ATATGCGGCCGCATGCATCTGCTGCTGTTGC | Clone Fs00219 to PVX for expression in N. benthamiana |
| Fs00219-Sal I R | CATGTCGACCTAAACTACACTCCTACTATCATTCATAAAG |  |
| Fs00341-Not I F | ATATGCGGCCGCATGATGCGGCTACTGCGC | Clone Fs00341 to PVX for expression in N. benthamiana |
| Fs00341-Sal I R | GTACCCGGGTTAAAGAGACGTTTCCGACCTAACC |  |
| Fs04405-Cla I F | GGCATCGATATGAAGTGGATTGTGCCTTTTCTG | Clone Fs04405 to PVX for expression in N. benthamiana |
| Fs04405-Sal I R | CATGTCGACCTACAAAGACCTATCGCTCGGAC |  |
| Fs05448-Not I F | ATATGCGGCCGCATGATTATCTCGAGGGTTTTGTTCCC | Clone Fs05448 to PVX for expression in N. benthamiana |
| Fs05448-Sal I R | CATGTCGACTCATAATACATATTTCCCGCAGCCAG |  |
| Fs06761-Cla I F | GGCATCGATATGAAGGCTACTTTCTTCCTCGC | Clone Fs06761 to PVX for expression in N. benthamiana |
| Fs06761-Sal I R | CATGTCGAC TTACAGAGCAAGGACAGCGC |  |
| Fs08184-Cla I F | GGCATCGAT ATGAAGCTGTCGGTGTTCACATC | Clone Fs08184 to PVX for expression in N. benthamiana |
| Fs08184-Sal I R | CATGTCGAC TTAAAGGGCAAACAGCATAGCCAT |  |
| Fs08420-Not I F | ATATGCGGCCGCATGACGCTCGGCAGGCG | Clone Fs08420 to PVX for expression in N. benthamiana |
| Fs08420-Sal I R | CATGTCGACTTACACTTGGGTATCTCCATCGCTTG |  |
| Fs08483-Cla I F | GGCATCGATATGAAAGGGCCTATTTCCATCCAC | Clone Fs08483 to PVX for expression in N. benthamiana |
| Fs08483-Sal I R | CATGTCGACTTACAGTAATTGCCAAAACACTGCAG |  |
| Fs09112-Cla I F | GGCATCGATATGAGTTCATTTACAAGCATAATAGTATTTGTG | Clone Fs09112 to PVX for expression in N. benthamiana |
| Fs09112-Sal I R | CATGTCGACTTATGTCCTTTCTACTGGCAGCTC |  |
| Fs09744-Cla I F | GGCATCGATATGGTCTTTTTGATCCGGTCGG | Clone Fs09744 to PVX for expression in N. benthamiana |
| Fs09744-Sal I R | CATGTCGACTCATACAGGCTCAGACGGAACA |  |
| Fs09882-Cla I F | GGCATCGATATGCATCCGTTGCTTTTGCT | Clone Fs09882 to PVX for expression in N. benthamiana |
| Fs09882-Sal I R | CATGTCGACCTATGCTAGACTACTTCTAGTTTCATTGG |  |
| Fs10166-Cla I F | GGCATCGATATGGCCAACAAGAACGCAAAGTCC | Clone Fs10166 to PVX for expression in N. benthamiana |
| Fs10166-Sal I R | CATGTCGACTCATCGAGGAGCAGCTGGCTT |  |
| Fs10591-Cla I F | GGCATCGATATGAAGCTATTCGCGATATTGCG | Clone Fs10591 to PVX for expression in N. benthamiana |
| Fs10591-Sal I R | CATGTCGACCTATCTGCATGTCCCTGTCTCG |  |
| Fs10692-Cla I F | GGCATCGAT ATGCAGTTCTCTACCTCTTTCCTC | Clone Fs10692 to PVX for expression in N. benthamiana |
| Fs10692-Sal I R | CATGTCGAC CTAAGCACAAGCAGTGCAGAC |  |
| Fs10706-Cla I F | GGCATCGATATGAAGTCCTTCTTCCTTTTCACCAC | Clone Fs10706 to PVX for expression in N. benthamiana |
| Fs10706-Sal I R | CATGTCGACTTACATGAGAGCGAACAAGAACATCAC |  |
| Fs11603-Cla I F | GGCATCGATATGCCCAAGTTCTTCTGCGATTAT | Clone Fs11603 to PVX for expression in N. benthamiana |
| Fs11603-Sal I R | CATGTCGACTCATCGCTTCTCATGTCCGG |  |
| Fs12429-Cla I F | GGCATCGATATGAAGTACTCTTTCGCTATTGCTGC | Clone Fs12429 to PVX for expression in N. benthamiana |
| Fs12429-Sal I R | CATGTCGACTTACAGAGCAAGGGCCATGAG |  |
| Fs13146-Cla I F | GGCATCGATATGGCCTCCCGCATTCTC | Clone Fs13146 to PVX for expression in N. benthamiana |
| Fs13146-Sal I R | CATGTCGACCTATATCAACAGAGCTCCCCAGG |  |
| Fs13485-Not I F | ATATGCGGCCGCATGCTCGTACCTCGAGTATTATATGC | Clone Fs13485 to PVX for expression in N. benthamiana |
| Fs13485-Sma I R | GTACCCGGGTCATTTTGACAAGAGCACTTCTTCTG |  |
| Fs13617-Cla I F | GGCATCGAT ATGAAGTCTTCTTTCCTCACCATCT | Clone Fs13617 to PVX for expression in N. benthamiana |
| Fs13617-Sal I R | CATGTCGAC TTAGAGGAGGAGCATGGCAATAC |  |
| Fs06761-NSP-Cla I F | agcaccagctagcatcgat atgCAGGACTTTGGTGGTCAACCC | Clone Fs06761-NSP to PVX for expression in N. benthamiana |
| Fs06761-NSP-NotI R | atcgtatgggtacgcggccgc CAGAGCAAGGACAGCGCC |  |
| Fs08184-NSP-Cla I F | agcaccagctagcatcgat atgCAGCAAGAGAAGTTACCAACATGC | Clone Fs08184-NSP to PVX for expression in N. benthamiana |
| Fs08184-NSP-NotI R | atcgtatgggtacgcggccgc AAGGGCAAACAGCATAGCCAT |  |
| Fs10706-NSP-Cla I F | agcaccagctagcatcgat atgCAGTCGAATTCCCCGGCTG | Clone Fs10706-NSP to PVX for expression in N. benthamiana |
| Fs10706-NSP-NotI R | atcgtatgggtacgcggccgc CATGAGAGCGAACAAGAACATCAC |  |
| Fs13617-NSP-Cla I F | agcaccagctagcatcgat atgCAGAGCTCTGATGATCTCCCC | Clone Fs13617-NSP to PVX for expression in N. benthamiana |
| Fs13617-NSP-NotI R | atcgtatgggtacgcggccgc GAGGAGGAGCATGGCAATACC |  |
| Fs06761-EcoR I F | CCGGAATTC ATGAAGGCTACTTTCTTCCTCGC | Clone Fs06761 signal peptide to pSUC2 for yeast signal trap assay |
| Fs06761-Xho I R | CCGCTCGAG AGCGGCGACGAGGCC |  |
| Fs08184-EcoR I F | CCGGAATTC ATGAAGCTGTCGGTGTTCAC | Clone Fs08184 signal peptide to pSUC2 for yeast signal trap assay |
| Fs08184-Xho I R | CCGCTCGAG AGCGGCAACAAGGCC |  |
| Fs10706-EcoR I F | CCGGAATTC ATGAAGTCCTTCTTCCTTTTCACCAC | Clone Fs10706 signal peptide to pSUC2 for yeast signal trap assay |
| Fs10706-Xho I R | CCGCTCGAG CGCCAAAACAGACGAGGAGAG |  |
| Fs13617-EcoR I F | CCGGAATTC ATGAAGTCTTCTTTCCTCACCATCTTCG | Clone Fs13617 signal peptide to pSUC2 for yeast signal trap assay |
| Fs13617-Xho I R | CCGCTCGAG GGCAACGGCGGCAG |  |
| Fs06761-PYBA-F | cgctctagaactagtggatcc ATGAAGGCTACTTTCTTCCTCGC | To amplify CFEM-ORF for complementary vector with GFP tag |
| Fs06761-PYBA-R | gataagcttgatatcgaattc CAGAGCAAGGACAGCGCC |  |
| Fs08184-PYBA-F | cgctctagaactagtggatcc ATGAAGCTGTCGGTGTTCACATC |  |
| Fs08184-PYBA-R | gataagcttgatatcgaattc AAGGGCAAACAGCATAGCCAT |  |
| Fs10706-PYBA-F | cgctctagaactagtggatcc ATGAAGTCCTTCTTCCTTTTCACCAC |  |
| Fs10706-PYBA-R | gataagcttgatatcgaattc CATGAGAGCGAACAAGAACATCAC |  |
| Fs13617-PYBA-F | cgctctagaactagtggatcc ATGAAGTCTTCTTTCCTCACCATCT |  |
| Fs13617-PYBA-R | gataagcttgatatcgaattc GAGGAGGAGCATGGCAATACC |  |
| Fs06761-NSP-PYBA-F | cgctctagaactagtggatcc atgCAGGACTTTGGTGGTCAACCC | To amplify CFEM-ORF-NSP for complementary vector with GFP tag |
| Fs08184-NSP-PYBA-F | cgctctagaactagtggatcc ATGCAGCAAGAGAAGTTACCAACATGC |  |
| Fs10706-NSP-PYBA-F | cgctctagaactagtggatcc atgCAGTCGAATTCCCCGGCTG |  |
| Fs13617-NSP-PYBA-F | cgctctagaactagtggatcc atgCAGAGCTCTGATGATCTCCCC |  |
| Fs06761-qRT-PCR F | CCCATCATCACCCCTTGCTT | qRT-PCR analysis in F. sacchari |
| Fs06761-qRT-PCR R | GCGGTCTTAGCGAACTCCTT |  |
| Fs08184-qRT-PCR F | GGACTTCCTCTCTGGCATCG |  |
| Fs08184-qRT-PCR R | GTCGGGAAGATCGGTCACTC |  |
| Fs10706-qRT-PCR F | TCCTGAATGCGCTCAACCTT |  |
| Fs10706-qRT-PCR R | ACGTAGGATTCAGCGAACCC |  |
| Fs13617-qRT-PCR F | ACTGCTCTGCTGCTATCTGC |  |
| Fs13617-qRT-PCR R | GGTGCTAAGGACGGTTCGAA |  |
| Fs Actin-qRT-PCR F | GAGAACGAGCGTGTCTTGATTGAGCC |  |
| Fs Actin-qRT-PCR R | TTTCCTCCGCAGAATGAAGAAGGACTC |  |
| NbEF1a-qPCR-F | TGGTGTCCTCAAGCCTGGTAT | RT-PCR analysis in N. benthamiana |
| NbEF1a-qPCR-R | ACGCTTGAGATCCTTAACCGC |  |
| Fs06761-AF | GACATTGACTGCTTTGATGTTGTCC | Amplification of upstream fragment of Fs06761 |
| Fs06761-AR | TCTTTCTAGAGGATCCCCGGGTACCTTGAATGTTACTGTAGGTAAATGATAATAAACG |  |
| Fs06761-BF | ATATCATCTTCTGTCGACCTGCAGGGAGACGGGAGTTCTGATGCC | Amplification of downstream fragments of Fs06761 |
| Fs06761-BR | CCATGACATGGCGCTTCATG |  |
| Fs08184-AF | CGCATGCAACCTGCCAC | Amplification of upstream fragment of Fs08184 |
| Fs08184-AR | TCTTTCTAGAGGATCCCCGGGTACGATGGATACTCGGACAGGAAAGATG |  |
| Fs08184-BF | ATATCATCTTCTGTCGACCTGCAGGGGGACAATGGCCGTGGC | Amplification of downstream fragments of Fs08184 |
| Fs08184-BR | GCAGGGCTATGCATGTACATAC |  |
| Fs10706-AF | GCATGAATATATGGGATAAGCCAAACC | Amplification of upstream fragment of Fs10706 |
| Fs10706-AR | TCTTTCTAGAGGATCCCCGGGTACTTTGGAACTGGTTGTAAGTTTGTATCGAT |  |
| Fs10706-BF | ATATCATCTTCTGTCGACCTGCAGGAAAAAAACAACATTCATTCACCAAGAAACG | Amplification of downstream fragments of Fs10706 |
| Fs10706-BR | GTTCGCCGGCAGCACAA |  |
| Fs13617-AF | GTGCCATCGGTGGTCTCC | Amplification of upstream fragment of Fs13617 |
| Fs13617-AR | TCTTTCTAGAGGATCCCCGGGTACCTTGATGGTTTTTCTTGATCGAGGATC |  |
| Fs13617-BF | ATATCATCTTCTGTCGACCTGCAGGATCAGGATGGACGAAAATTTGATGTTT | Amplification of downstream fragments of Fs13617 |
| Fs13617-BR | CCCGCTCGGCTAGGTATG |  |
| Hyg-F | CGGTACCCGGGGATCCTCTAG | Amplification of hygromycin resistance gene |
| Hyg-R | GCCTGCAGGTCGACAGAAGATG |  |
| Fs06761-F1 | GTTCCGAGAGTGGCTGCC | Knockout transformants PCR verification |
| Fs06761-R2 | GGTCCTCCGCAAGGCATC |  |
| Fs08184-F1 | GCATGAATTGAGACGCTAAAGGTTC |  |
| Fs08184-R2 | TAACGAATCCCTAGCTTATGGTGC |  |
| Fs10706-F1 | GAGCTTTAATTGAGTTCAGTTCATGATG |  |
| Fs10706-R2 | GCGTGCGCCATGATGAAG |  |
| Fs13617-F1 | GCCCGTGCCGTGCTTG |  |
| Fs13617-R2 | GGCAGAGTGAGATTGGCAAGT |  |
| Hyg-R1 | CGCCCCAGCACTCGTCCG |  |
| Hyg-F2 | GGCCGCAGCGATCGC |  |

**Table S2. The nucleotide sequence of TEF-1α, RBP1 and RBP2**

TEF-1α (GenBank accession numbers: MK829752)

TCGTCATCGGCCACGTCGACTCTGGCAAGTCGACCACTGTGAGTACTACCCTCGACGATGAGCTTATCTGCCATCGAAACCCTCACCAAGACCTGGCGAGGTATTCCTCTTGAAACAAGATGCTGACATGGCTACACAGACCGGTCACTTGATCTACCAGTGCGGTGGTATCGACAAGCGAACCATCGAGAAGTTCGAGAAGGTTAGTCACTTTCCCTTCGATCGCGCGTCCTTTGTACATCGATTTCCCCTACGACTCGAAACGTGCCCGCTACCCCGCTCGAGACCAAAAATTTTGCGATATGACCGTAATTTTTTTGGTGGGGCATTTACCCCGCCACTCGAGCGATGGGCGCGTCTTTGCCCTTTCCTATCCACAACTTCAATGAGCGCATCGTCACGTGTCAAGCAGTCACTAATCATCTGACAATAGGAAGCCGCTGAGCTCGGTAAGGGTTCCTTCAAGTACGCCTGGGTTCTTGACAAGCTCAAGGCCGAGCGTGAGCGTGGTATCACCATCGATATTGCTCTCTGGAAGTTCGAGACTCCCCGCTACTATGTCACCGTCATTGGTATGTTGTCGCTCATGCCTTACTCTATTTCCTAGTACTAACATGTCACTCAGACGCTCCCGGTCACCGTGACTTCATCAAGAAC

RPB1 (GenBank accession numbers: MK983417)

GGAGCAAATCATGAACTGTATGCTCTGGGTTCCTAACTGGGACGGTGTCATTCCTCAGCCCGCCATCTATAAGCCTCGTCCTCGGTGGACTGGTAAGCAGCTCATCAGCATGGTTATTCCTAAGGAGGTTAGCCTGTTCAACGGTACGGATTCTGGTGAAAATGCCCCTCTTAAGGACGAGGGTCTTCTGATCCAAGCCGGCCAACTGATGTATGGTCTTTTGACGAAGAAAAACATTGGTGCTGCTGCCGGCGGTATTGTGCATATCAGCTACAACGAACTTGGGCCCGAGGGTGCCATGGCTTTCTTGAACGGTGTCCAGCAGGTTGTCACCTACTGGCTTCTCAACAATGGTCATAGCATTGGTATTGGTGATACAATTCCCGACGCGGCGACCATTGCTAAAGTTCAGGTACATATTGATGAGGAAAAGGCTGAGGTCGCTCGCTTGACAGCAATGGCCACAGCGAATGAGCTTGAGGCCCTACCAGGTATGAACGTTCGTGCAACCTTCGAAAACAAGGTCTCCATGGCTCTGAACCAGGCCCGTGATAAGGCTGGTACCACAACACAGAAGAGTTTGAAGGATTCAAACAACGCTGTCACAATGGCTTCCTCAGGTTCCAAGGGTTCATCTATTAATATTTCTCAAATGACTGCGCTTGTCGGTCAGCAAATTGTCGAAGGCAAGCGTATTCCTTTTGGTTTCAAGTATCGCACACTGCCTCACTTCACCAAGGATGATTACTCACCTGAGGCCCGTGGCTTCGTCGAGAACTCTTACCTCCGTGGTCTCACTCCCAGCGAATTCTTCTTTCACGCCATGGCTGGTCGAGAAGGTCTCATTGATACTGCAGTCAAGACTGCCGAAACAGGTTATATTCAGCGCCGATTGGTCAAGGCTCTGGAAGATCTTTCTGCCCGTTATGATGGAACTGTCCGAAACTCTCTGGGAGACATTGTTCAATTTCTCTATGGTGAAGACGGTCTCGATGCCATGATTATTGAGAAGCAGAAGCTGGGTATTCTCAATATGTCAAACTCGGCATTTGAAAAGAAGTATCGCCTGGATCTTGCCAATCCCCCGGATTGGTTCAAGTACGACTACGAATTCGGTAACGAATTGACTGGCGACAAGGAATCTATGGAGTATCTCGATCAAGAATGGGAAAAGCTGTTGGCTGATCGTCGACAAGTGCGGCAGATCAACAAGGCCAAGGGAAACGAGGAGATGATGCAGCTTCCCTTGAACATCACTCGTATCATCGAGTCCGCTAAGCGAGTTTTCAATGTCAAGGCCAATGACAGAAGCAACTTGCGACCATCAGAAGTTATTCCAGCCGTGCAAAACTTGCTGGATAGCATGAAGATTGTTCGTGGTACAGATGAAATCTCGGTTGAAGCTGACGCAAATGCATCCATTCTCTTCAAGGCCTTGCTTCGCTCTCGCCTGGCTTTCAAGGAGGTGGTCAAGGAGCACCGGCTGAACAAATTGGCTTTCGACCATATCTTGGGTGAACTCCAGAATAGATGGGATCGCGCATTTGTCAACCCTGGTGAA

RPB-2 (GenBank accession numbers: MK829720)

CAATACTCCCATTGGACGAGATGGTAAATTGGCCAAGCCTCGTCAGCTTCACAATACACACTGGGGATTGGTGTGCCCTGCCGAAACGCCTGAGGGTCAAGCTTGTGGTCTGGTCAAAAACTTGTCTCTGATGTGTTATGTCAGTGTCGGCTCTCCAGCCGAACCTCTTATTGAATTCATGATCAACAGAGGTATGGAAGTCGTCGAGGAGTACGAGCCGACAAGATATCCCCACGCCACAAAGATTTTTGTCAACGGTAGTTGGGTTGGTGTTCACGCCGACCCCAAGCATCTCGTGAATCAGGTTTTGGACACAAGACGAAAGTCATACGTCCAGTTCGAAGTATCACTTGTTCGTGATATTCGAGACCGTGAATTCAAGATCTTCTCAGATGCCGGTCGTGTTATGAGACCCGTCTTCACAGTCCATCAGGAGGATGACTATGAGAACAACATCACTAAGGGACAACTAGTGTTGACGAAGGAACATGTCAATAGGCTAGCCCAAGAGCAGGCAGAGCCGCCTGCCAACCCCGCTGACAAGTTTGGGTGGGATGGTTTGATTCGCGAAGGAGCTGTCGAGTATCTCGACGCCGAAGAAGAAGAGACAGCCATGATTTGCATGACGCCAGAGGATCTCGAACTCTACCGCGAGCAAAAGAATGATGAAGCTACGCTTACGGAGGAGGAGAAACGAGCCAAGGCTGAGGCAGAGAAGAGGGAACAGGAAGAGGACCGCAACAAGCGGTTGAAGACAAAGGTCAACCCCACAACTCACATGTACACACATTGTGAGATTCACCCCAGTATGATTCTCGGTATCTGTGCCAGTATCATTCCTTTCCCCGATCACAACCAGGTATGTATGTCCTNNNNNNNNNNNNNNNNNNNNNNNNNNNNNNNNNNNNNNNNNNNNNNNNNNNNNNNNNNNNNNNNNNNNNNNNNNNNNNNNNNNNNNNNNNNNNNNNNNNNTCGTTCTATGGAGTTCCTCAAGTTCCGTGAGTTGCCAGCTGGTCAAAATGCCATTGTCGCGATTGCTTGCTACTCGGGTTATAACCAGGAAGATTCCGTTATTATGAACCAGAGTAGTATCGATCGAGGTCTGTTCCGAAGTCTGTTCTTCCGATCGTACTCAGATCAAGAAAAGAAGGTTGGTCTCAACTACACTGAGATCTTTGAGAAGCCTTTCCAGCAGACAACACTTCGCATGAAGCATGGAACATACGACAAGCTTGATGAGGATGGTATCGTGGCGCCTGGTGTCCGTGTGTCTGGTGAAGATATCATTATCGGCAAAACTGCACCCATCGACCAAGAAAACCAAGACCTTGGCACAAGAACTCAATCGCACCAGCGTCGTGATATCTCGACACCATTGCGAAGCACTGAGAACGGTATCGTTGATCAAGTCATTCTGACAGTCAACGCCGACAACGTCAAGTACGTCAAGGTTCGAGTACGAACCACCAAGATTCCTCAAATTGGTGACAAGTTTGCTTCTCGTCACGGTCAAAAGGGTACAATCGGTGTTACATATCGACAGGAGGATATGCCTTTCAGCCGAGAGGGTCTCACTCCCGATATCATTATCAACCCTCACGCCATTCCATCGCGAATGACAATTGCCCATTTGATTGAGTGTCTTCTTAGCAAGGTTTCAACACTGGAGGGTATGGAGGGTGACGCTACACCATTTACTGATGTCACAGTCGATTCAGTTTCTGAACTTTTGAGGAAGCATGGTTACCAATCTCGAGGTTTTGAGGTCATGTACAATGGTCACACGGGACGAAAGCTCCGTGCCCA

**Table S3. The nucleotide sequence of 20 CFEM genes**

>FSAC 00219

ATGCATCTGCTGCTGTTGCTGGTGACGTGGGCGTGTCTCGTATGCCAAGTGTTCTGTGACGATGTCTCAGACTTCCTGACAAAGATACCAGACTGCGCTGGGAGCTGTCTTGTCGAGCTTGTATCAACGTCGACTTGTGGCATTGACGTACAATGTCTTTGCGCTGACCCGAAACTCAAGACGCAGGTTTTAAGCTGTGTTCAGAAGAAATGCCTCCCGAGAGACGCATTAGCGACGCTGAATGTCACCTCAGTTGCGTGTGATTTTCCGGTACGAGACAAACATGCGCAATTCGACATTTTGGCCATCACGCTCATAGCAATCACTGCTATTGTGGTCGGTCTTCGACTTTGGCACAAGCTGCGATACGAAAGGAAGTTTAGGCGAGATGATTATCTTGTCGTAGCGGTCTTTCTGCTTGATTTGGGAAACACGATTGTTTGCGTTCATGGCCTTTCGGGCAATGGCCTTGGGAAAGATGCATGGCTTTTCAGCCCAGACACGATCAACAGCTTTCTCTGCTACATCTACATTGGCCAAACCCTCTACGCATCCGATGTTTTCCTGACCAAGATATGCGTCGCCCTCTTCTATCTCCGAATCTTCCCCGTCGTATCCGTCCAACGTCTTATATGGGGTACCATCATTTTTTCGGTCCTAGGCATGGTCATCTTTGACATCCTTGCTATTGCACAGTGTCAACCGATCAGCTTCTACTGGACAGGATGGGATAAGCTACATGAGGGACATTGTCTTGGAGTTCAGCCGTTAGCTTGGGCCATCGCCGCCGTCGGTATCATCCTGGACGTTTGGATGTTGGCCATTCCTATCTGGCAACTGGTGCAATTGCAAATGAAGTGGCAAAGGAAGCTCGCTGTAGCTATCATGTTTACGGTCGGAACTTTTGTCACCATCGTCAGTATTTTGCGACTCCGGTACTTGGTTGCTTTTGGAAACTCTCAGAACCCAACCTGGGACAGTTTTGAGACCTGTTACTGGTCCGTTATTGAGATTAACGTTGGTCTCTGGTGTGTCTGCCTTCCCGACCTCCGGTTGCTTATCCTCAAGGCATTTCCGAGACTTGGAAGTTCTATCGATTCAGGTCCTAGAAATCCCCACCAAGGATCATCGGCACCCAGGCAGCAGGGCAGAACTCCGAATTCCTCTCGTCATACCGAGAGTACTATTTACAAGGGACAGCCGATTCAGGCCCAACAGGAAGCTTCATCGAGCACAGCTGAGCTTGTTGAGATGACCACCTTTATGAATGATAGTAGGAGTGTAGTTTAG

> FSAC 00341

ATGATGCGGCTACTGCGCGGGGCTGTCTTCGCTTGCCTTGCGGCTCTGCCGTTGGCTTTTGCTCAGCAAGAGTCTTCCTCTTCTTCTTCTGCTGCTTCTGCTTTGTCTGTGTTACCGGAATGTGCTGCAAATTGTTTCGTGTCGGCCGTTGAGCGATCGACATGTGAACTTACAGACCAGAAGTGCATGTGCACTAACACCGCACTACAACAAGACATCGAAGGCTGCGTCATGCGCGCATGTACAATTCCACAGTCACTTGTCACGAAAAATGCTACCCTCACAGCATGTGGCGCTCCAGTCAGAGACCACTCACCCCAATTCGTAGTCCTCAATGAAGTCATGGCCATCATCACGGGAATTTTCATAATCCAACGCTTTGGTACAAAGCTCTACTGGAAGCTCCCACTCGGCCTTGACGATCTCTTCATTGCACTGACAATGTGTGTCGCCATTCCTTCCATCGTCATCAACTCTCGTGGTCTCGCTCCCAACGGCATGGGTCGCGACATCTGGACTGTTACACCTGATCAGATCACCCATTTTGGAATGTTCTTCTACACCATGGCCATCCTGTACTTCTCACTGCAAACGTTTCTGAAGTTGTCCATGCTGTTCTTCTACCTGAGAATCTTTCCGACACAGAATGTCAGGAGATTGCTTTGGGGAACTGTTGCCTTCACTGTTGTCTTTGGGCTGGTTTTTATCTTCGTAGCCATCTTCCAGTGTCGACCTATCAACTACTTCTGGCTGAAGTGGGATGGACAACATCAAGGCAAATGTGCCGATATTAACGCGGTTACTTGGTCCAATGCCGCTATCAACATTGCCCTTGACTTTTGGATCCTTGCCATTCCCTTGTCTCAACTCAAGTCACTAAACTTGGATTGGCGAAAGAAGATCGGTGTTGGCATGATGTTCAGCGTCGGTATCTTCGTCACAATCATGAGCATTCTTCGTCTTCACGCCACAGTCCAAGCCGGCGTCAAAACCTCCAACGCAACATGGGAATACCTCGCTGTATCAAAGTGGTCAACCATCGAAGGCAACGTCGGTATCATCTGCGCCTGCATGCCTTCCCTTCGAATTCTTCTCGTCCGCCTGTTCCCCAAGATTCTTGGTACTTCCCAACGCTACTACAACTATGGCAGCAAGAGCAACAAGCAAACACCTGGCAATACTCACAATCGCAGTATTCCTCTTGGAACAAATGCTACTTCTCAAGCGGATCGATCACAAAGAAGGGTTGACCCTATCGGCATTGAATGTCATAGGACATATGAGGTGGAATATGGAGACAATGACGAGACATACTTGGTTCATATGAAGGATATGGATCATAAGAGTGCAAGGTCGGAGGTTAGGTCGGAAACGTCTCTTTAA

> FSAC 04405

ATGAAGTGGATTGTGCCTTTTCTGGTGGCCTCTGTGCCCCTGGCAGCGGCAGCGGATGCAACCAAAGAGTTGTTGACCAATCTGCCTGCCTGTGCAGCTCCTTGTTATGAAGATGCCATCAAAAAGTCGGACTGCAACTCGACCGACGTGAAATGCGTGTGCTCAACTCAAGTCATCATCCAAACAGCAGAGGCATGCGCCGCCCAAGCATGTCACGTCAGAGATAGTCTGACAACGCTCAACATGACATACACCTACTGCGAAGTCCCCGTGCGAAACAAGACCCCCATCTTCATCAACGTCACAATCGTCCTCGGCGTGATATCCGGCGTCGCGACCGCCCTGCGCCTCTGGTCCAAATTCTTCTTCACCAAGACCGAACTCGGCCTCGACGACCTCTTCATCGTGCTGACGCTCTTCATCGGCATGCCCTCCACCGCCATGAACATCCACGGAACCGCCGGGCACGGAGAAGGCCGCGATATCTGGACGCTAGAGTTTGATGACATCACAAAGTTTGGTTTCTACTTTTGGTTGCTCGAGATATTCTACTTTGCGCAGGTTTCGCTGCTCAAGACGTCCTTGTTGTTCTTTTACCTGAGGATCTTCCCGGGGAATGCGCAGAAGTTGCTCTGGGGGACTATTATTTTCAATAGTGTGTTTGGTGTCTTGTTTATGTTTTTGGCGGCGTTTCAGTGTACGCCTGTGAGTTATTTCTGGTTGAATTGGGACGGGGAGCACAAGGGAACATGCATGAACTCGACTGCCATCGGCTGGGCCAATGCGTCTATCAGTGTTGCTGTGGACGTGTGGATGCTTGCAGTTCCATTGTGGTATCTCCGAAAACTCAAGCTGCATTGGAAAAAGAAGATTGGTGTCGCTGCCATGTTTATCGTCGGAACTTTTGTCACCGTCGTAAGCATCATTCGTCTCCAATTCCTCGTTGATCTCGGCACGTCTCGCAACCCAACATACGACCAAACCGACATCTCCATCTGGTCAACCGTTGAAATCAACGTCGGTATCATCTGCGCATCTATGCCGGCTCTTCGAGTCATTCTCGTCCGTCTGTTCCCCTCTCTCGGAGGCTCATCCTACGATTCGAGCAAGTACAACAACTACGGAGAACATTATGGACGGAAATCACATATCATGAGTCGAAGTCGTGCTCGAGTGGAGTTACCTTCTCACACAGGTGACTCTATACATACGCCTGAGCATGGAGGTATTGAGTTGCAGAGGACGTTTCACGTGCAGTATAGTGAGAATGATGAGCAGAGTCTTGTTAATGGGGAGAATAAGTTTAACAAGACGCAAGTTACGACTCAGATGAGGTCTGAAAGTCCGAGCGATAGGTCTTTGTAG

> FSAC 05448

ATGATTATCTCGAGGGTTTTGTTCCCCCTCTGTTTGGCAGTGTTGGCCTTGGCTATCTCACTGTCAGAGGTACCGTCATGTGCAATTCCATGTATTCTCCAAACGATGGACTCTTCGGGCGAAACGAATGCGAATATAAGATCGATGTGCAATGACCCGGGCTTTCAGACCGATGTGCTGAATTGTGTTACAGGGGTATGCACTGCGCAAGAGATACAAGGCTTCATCGTAATGGGAAAGGAGTTATGCCAGATGCCGCTGCAGGACAATCGACAAGAGTATCGGGCCACGATCATTGTGTTCGCAACGTTGTCATTCTTCTTCTTCGTGTTGAGGGTAACGTCCAAGATGGTTACCAAGAATACATGGGGCACTGATGATACCTGGGCAGCTATCACTTTTTTCCTTCTGATACCATTTACGGTGTTCACGCTCCTCGCAATACATCACGGCCTCGGACTCGCTACGCCACTGTTCACCAAAAATGACCTCTCCAAAGCTCTGAAGGAGATCTTTATCCTTCACCTTCTCTACGTCTGTGGTCTCGCAGCAGCCAAGACATCGATTCTTTTCTTTTATCTACGTATTTTCTCAGACAGTTCGTTCAGAATGTTGGTCTGGGTAACCCATGCCTTCAACGCCTCATCGACTGTCATCATTATCACCCTGAACCTTACGCTCGGGCGGTCTGTCACATATCTCCTCGACAACAGCTCAGATGCCGGGGTGAACATGAAAAAATACTCGAATGCCCTCAAGATTGTTCTGGCGCACTGCGTGGTAAATCTGGCACTTGATATTTGGATGCTCATCCTTCCAATGACGCAGCTATACAACATTGGGCTGAAGCTGAACAAGAAGATCAGCGTGATGGCCATGTTTGGTCTTGGTCTGTTTCTCACGGTAGTCAGTCTGATTCGGACGATTTACCAGTCGCAGCTCCTTGCCAAGCCCGAAGAAGCGCTCGCCAGCGTTCCTTCAACACGCCAGCTTATTCGGAAAGCTATCTATCAGATGAGGAAGCAGAAAGAAGGACAGTTAACAAACAAACCTATTTTCATTGACAGGTCATTGGTGCCGATACCGGATGAAGAGGACATGCCGACGCTGAGTGACGTTGGGGGACTGACGAGTGTGACAGTCAGCAGTGGGCGAACGGCAGAACCAGAAAACTTGGCACATCGCGATGCAACTGACGTCACTGGCTGCGGGAAATATGTATTATGA

> FSAC 08420

ATGACGCTCGGCAGGCGGGTTCTCATCTTGGGGACGATATGCCTGCTGTATGGCACAGCGTCGGCTCAATCTATGCTCGCAGAAGCTCCAAAATGTGCTATTGATTGCCTGACCGAGCTTCTGAGCCAGAAGGAATATGCCGAGTTGGGACAGGAGGCAATGTGTAGTAGCAAGCCGTTCGCCAAAGCGATGGGTGTTTGTCTGATGGTCAAGTGCTCTATGAGACAGACCATGGACTTCATCAAAGACTCGTCAGCTGCATGCGGGATCCCGCCAACCAACAATACAACGTCCTATCGAGTAAACAGTACCGTCGTCTTTGCATTTGCGCTCGTATTCTTCGCCTTGAGGATAGTAACTAAATTCCGGCTTGGCCTTACCTGGGGGATTGACGATACCTTGACGACTTTATCGGTTGCAGTTATGATACCTTATTATATCGTGTTGCAGATCATGCTTGCTTTGGGACTTGGTCTTGATATGTGGTTCATAAGTGACAGTCAGATCATACTGATTTTCAAGTTATTCATCGTCATTGAAGTTTTGTACTTGACAGCACTCGTCCTCGTCAAAGCAGCAATCCTGTGCTTCTTCCTCCGGATCTTTCCCGACCACAAGTTCCGAATTGTAGTCAAGTGCACCTTGGTCTTCAACGCTCTGATCTGGGTAGGTTTCTTCATCTTCGTCTTCTTCCAAATACAGCCGTTTTCGCTGTTCTGGAACGGTTGGCAGCAGAAGAAGGGCCATCTTATCCTGACCGGGTTCACCAATTTCACTCTGCCGCTCGCGGGGATAAATCTGCTGCTGGATATCTGGATGCTCATCCTGCCCATGACGCAGCTCTGGGGGATGGGGTTGAAGCTTAAGAAGAAACTCGGTGTCATTAGCATGTTCAGTGTTGGTATATTTCTTACAATCGTCGCTGTGATTCGAGTTCATGAGCTTGTAGCCTTTCTACTCTCTCAAGACTTAACGGTCGATCACGCACAATCAGCCTTCATCTGGTCAAACGTGGAAATTTCAGTCGGTGTAATGGTCGCTTGCATGCCGCACATTCGACATCTGGTGCGACATATAATATCGCGAATAAGAGCAAGGAAGGGAACAGAGCCGAGACAAAACAACAGAAAGATATTCGTCGATCGATCGCTTGCGACTATTGAGGTGGGGGAGTCGCAGGCCATTGAACTGAACGATGAAGGTGGACTCTTGACGGCCAATACGTGTACGACGGCTTCATCGGCGACAAGAGTCGGGACAATTTCGACAACAAAAGAGGGGAGAAAGGACAGTAAATCATATGCGACGGTCAGTTTTGCAAGCGATGGAGATACCCAAGTGTAA

> FSAC 09112

ATGAGTTCATTTACAAGCATAATAGTATTTGTGACCTGGTGCCATTGGGCTTCCATTGCAGCCGCTCAAGCTGAGTTTCCTAGTTGCGCTAGCACATGCTATTCTGGTTTAGTTAGCAGCAGCTCAACACCTACAACAAACTGCAACAGTTTCGACTACAAGTGTATCTGTCAAGATGAAGATTACCTCGAAGAGCAGATCTGCTGTTTACACAAGGAATGTTCCGACGGAGATCGCGAGGCTGCTGAGGGCTATGCAAGATCTGTCTGCCAAGCTGTTGGTGTGACTTTGGGCCATAACCTGACTTGTCCCACTCTGTCGGCATCTGCTTCAAGTACAGCCACAGAGGTTTCAGGAAGCAGCACCAAGACCGATGCTGCAGGCTCCTCTGCATCTTTAGACTTATCCTCTGGTAATAGTGGTGGAGCCGGGGATGAAAGCTCAAAGACTCACATAGCTCACCAAACATCGCAACATAAACTTAAAACGGGATTAGGCGCGGGCCTGGGAGCGGGGATACCATTCCTGGGCGCCATCATAGGTGCCATTACGTGGTTTTTAAAGAGGAAGAAACGCCAGTCTCTTACACAGACACCACACGCCAACACAACAGCTCAGCCTCCATTTGATCCCGCGGCCCCAACCCAAGAAGTTCAATATGTTCACCAGGCACCTAGTGGTTCATTTTACTCTATGCCTCAAACAGATCCTCAGACTACAGCATACTCGCAACAGCCAATATTTGAAGCTTATGGATCGCCTGGTAACAATAGACAAGAGCTATACGGTGGTACAAAACTTCCTGCTGCTCATGAGCTGCCAGTAGAAAGGACATAA

> FSAC 09744

ATGGTCTTTTTGATCCGGTCGGCAATGAAGGTCCTTGGTTCGCTGGCACTAGTGCTGGCTTTATTTCAGCTTGGTGTATCGGCCGCCGCGGCGCCCGAAGAGCCTCCAACTTGTGGTGCACTCTGCATCGAGGAAGAAGCGATCAAGTCGCCTTGTGGTCTGAACACTACCTGTATCTGTACAAATGTTGAGCTTAATGAGAAGATCTCGGTTTGCGTCGCGGCGAATTGTACGGTGCGGGAGAGTCTGTTGGTTCAGAGTTACTCCAAGCATACTTGCAAAGCGCCATCTCGCGATCGCACAACGCTGGTATGGGTTATCGGCATCGTCTTCTTAATCCTCGGCCTCATAGGTTTTGGTCTGCGTGTCATGGCCAGAGTGTTTGTCGTGAAGCAAACATGGGGATCCGACGACTGGGTTATGTTGTTTGCAGTGGCTCTTATGGTACCGCTCAACGCATTGTCTGTGCAGATATCGCGCGTGGGACTCGGCAAAGACATATGGAACGTTCATCCCGATGATATCACCGACTTCCTTTATCTATTCTACTGGGACGAACTTCTCTATCTCGGAGCTTTGCCCGTCACCAAGATCTCCATTCTTCTCTTCTATCTCAAAGTGTTCCCCGGCAAAAACATTCGCAGGGCATGCTGGGTCTTTATCGGTCTAAACGTCGCGTACTTTATCACGTTTGAGCTCATTTCCATTTTCCAGTGCAGGCCAATTGAGGGTGCCTGGAGAGCATGGGACAAGGAGTTCAAGGCCAAGTGCAACAACATCAACTTGCAGGGCTGGTTTGCGGCGATCCTCAACATTCTTCTCGATGTTGGAACGATGGTCATACCGCTGAAGGAGCTTTATGGGCTTTCGATGTCGCTCAAGAAGAAGATACAGCTCATGCTCATGTTCAGCGTCGGCATCTTCGTCACCATTGTCAGTGCAGTCCGCCTCCAATCTCTAGCAAGCTACGCAACAACCAGCAACGTAACCCAGGACTACGTCGAAATCGGCTACTGGAGCACAATCGAAGTGCCCGTGGGCATAATCTGCGCCTGCATGCCCGCCATCCGCGCGCTCTTCGGCATCGTCTTCCCCAAGGTCTTCGCCTCGACGAACCGCAGCAAGAACAGCTACGCCAACATCTCCAAGGAGTCGAAGCAGCCAGCATCCGACAGGAAGGGCGGCAACACGCCGCAGATCACCATCGAGACGGAGATCTCGACCAGGTACTCGCGCCACCACGACGATTCTTCGGTAATTGAACTTACGCAGATGGGGAGGGAGCAGGGCCACGAGGAGACTGCGTGGGCGGAGAGGAGGCCGGCTGTTCCGTCTGAGCCTGTATGA

> FSAC 09882

ATGCATCCGTTGCTTTTGCTGGCGACATGGGCTTGGTTCATGAGCCAGGTTCTTGGCAGCGATCTTTCCAACTTTTTGACTCAGCTCCCCAGCTGCGCGGAGAGCTGCGTCCTTGATTTTGTCTCATCGTCTTCCTGCGGGACGAACGCAACATGTCTCTGCACAGATCCAAAGCTCAAAGACGACGTGCTGCCATGCGTTGAGAGTCATTGTCTGCCCAGAGACGCCCTCGCGGCGATAAATTTGACCTCGGTGGCATGTGACTTCCCCATTCGCGACAAACACGAGCAGTTCAATATCCTCACCATCACGTTAATTGTGATTACTGGAGTCACAGTTGGGCTTCGGTTTATTGAAAAGATACGATATGGGCCTGGACTCCAGATTGACGACTACGTTATTACGGGCGCTTTTCTGGTCAATTTAGGCAACTCGATTGTCTGCTTGCATGGTCTCTCTAGAAATGGCCTTGGACGAGATGCGTGGAGATTTAGTCCCGACACAATCACATCCTACCTATGCTTTCTCTACGCTGGTCAAACTCTCTACGCCACAGACGTTTTCGCCACCAAGATCTGCGTTCTTCTCTTTTACCTACGCATATTTCCCGGTGTTGTCATTCGAAGGTTGATATGGGGAACAGTTGGAATGGCAGTGCTTTGCATGATCATCTTTGATCTGCTTGCACTTTTTCAATGCCAGCCCATTAGCTTCTATTGGAAGGGATGGGACCAACTGCATAAGGGCCATTGCATAGGGATCAATGGATTAGCATGGGCTATTGCTGCAGTGGGAATCATTCTAGATCTCTGGATGCTTGCAATTCCGATTTCTCAACTGATTCATCTCCAGATGAAATGGAAGAGGAAGCTTGCAGTTGCATCAATGTTCGGCGTGGGCACTTTTGTGACGGTTGTCAGCATCCTCAGGCTGCGCTATCTAGTAGCATTTGGCAATTCCTCCAACCCTACATGGGATAGCTTTGATACATGCTACTGGTCCATCATAGAACTCAATGTCGGTATCTGGTGCGCGTGCATGCCAAACCTTCGGGTTTTGATGCTCAAGACATTTCCAAGACTGCAAAGCTCAGTGGATGCTACTCCCAGAAGCCACCAATACAACTCGTCTACCGCAGGACGGCCGATAAGAGTGCCGAGCAATACGCCAGGTCTATCCGATAACACCATGTATAGAGTGAGATCTGATCAAGGGCGCAAGGTCGGCGGCTCATCAAGTACAGCGGAATTGGTCGAGATGACGAGATTCACCAATGAAACTAGAAGTAGTCTAGCATAG

> FSAC 10166

ATGGCCAACAAGAACGCAAAGTCCCTCTATGAGGCTATTCCGGAATGTATAAGTGGCTGCTTCGACATCAGTGTTGCTAATACCGGCTGTGCCAACGACGACTACGACTGCTGGTGCTATAAGCCGAATCATCAGACCATCGTCGATACGTTGGAGCAATGTCTCTCGAATAAAGAAAGGAAGACCAAAAAGAAGTGTACTGAAGATGAGGAATTCGAATATGAGAATAGTTACTGGAAGATCTGTGAACAATACTGGGAGCCTTATGGAACAGCCACCGAACCAACATCATTTCCAACTGCTGTATCGTCTACAGCCTCAGCAGCGTCAACAACACTTAAGGTCTCGACAACAGTCGCCACTACTGCATCGTCTGAAGAGACAGCCGCCGCGGAAGAAGCTACATGGACGAGCCTTGCACCAGCTGAGACTGGCGATTCCGAGCAAGCGCAAGCATCTGGAGAAGCAGACATAGTGACATCTACTCATAGCGGTCTCTCGCCAGGAGGGAAAGCAGGAGTGGGCGTTGGTGTTGCAATTGGTGTCATCTTAATCGGTATTGCCGTCTTCCTCTGGTTGCGGGAAAGGAATCGAAGACGCTCAGTCGAAGAACAGCTCAGAGTTGTTGAGATAGAGAAAGCCAACGCTTCTCAAGAAGGATATTATGTTAGCAAGGGATTGTACGAGATGGAGGGCGATCGGCCCCATGCGGAGGAACTCCGCGGATGTATGAGAACGCCAGAGCTTGGTGTAGGAGAGATGACAAAGTCAAGCAGCGTGACACACGTGGGACCTGTGAGTCCATCTGATGACGATAGGGACTCGTCGTTTTCGACACGGAGTCACTCGTGGCCCATTTCACCCGAGAGCCCAGGTCGTCAGGAGTCAAGAGGTTTGGGTGAGATAACGGACAATAATGCGGCCAAGCCAGCTGCTCCTCGATGA

> FSAC 10591

ATGAAGCTATTCGCGATATTGCGCTTGTTCATTCTCATTTTATCGTGTATATTCCAACAAGTCGGCGCTAGCTCACCGACCACTGCGCAACGGCTTGAAGACGCGTCGCAGTGTGCCATCAAATGTTTCTCGCAATTCGTTGATAAGCCGACGTTCTCGACCGCCAACAAGGAGCGGATATGCTATGATCACAAACTCAGCAACACGGTAGCCAGCTGTATCCACACAGCATGCCCGATACGAGACCTATTCGATTTTCTCAAGCTTGAGCAGACGAACTGCGGCAGACCAGAGCTCGACAACGATCACAACATTAGGGGGATCAACTACACGATCCTTGGGATAGCTGTGTTTTCTATCGTCCTTCGAACTGTCACCAAATCATATCGCTTTTCGCAATGGGGTGCTGACGATTACCTCATCATCGCGGCGTCGGTCTTTACTGGAGTTCAATGCATCATCATGGGCCTGATGACATTTGCAGGCTTGGGCCGGAATATCTGGACACTTGACGACAGTACCATTACCACGTTTCATATTTACCTACTCGTCGTCCAATACGCCTACGTTATAAGTCTGTGTCTCATCAAACTTTCAATACTCTATTTCTTTCTCCGAACATTCCCAGACCCAAAATTCAGATTGATCATCAAATGCACCATCGCCTTCAACATCGTCACGACAATCATCGTCATCATATGCGGCGCACTGCAGCGACAGCCAATCCACTTGCTCTGGGATGGATGGAAATATTATCCACCTCGCGGGACTACACTTAATACTCCGGCAATCATCTTCTTTCACGCTGGTGTCAACATTGCCCTTGACATCTGGATGTTTGCACTGCCATTGACGCAGTTGTATTCTTTGGGGCTTCAGCCAAAGAAGAAGGCTGGCGTTATGGTGATATTTGGCGTTGGGATATTTCTCATTGCAGCAAGCTGTATCCGCATTCCCTATCTACTCGACTTCACAAGAACGTTGAACGCATCATCCGACGCACAAGGCTTCGTCGTATGGTCCAATATCGAAAGTGGCGTAGGCATCTTGGTAGCCTGTATGCCTCACATGCAACCAATCTTCCGCGCCATAGCAGCACGAGCAAGGTCATGGAACATTCTACCCCAAGGCTCGAGCAGTAGCACAGAGGGAATATTTGTTCAAAGATCTCTGGCGACCATCAAGATGTCGCGGACTGATGGAACTACTTTGGTGGAACCGGACGACTTGGTGCTGCATGATACCGGGGGTTTGTTGAGCGAACCCAGCCCGGCGAAGGTTGATAGTAAGTTTGGGAGTGTGAGCGAGACAGGGACATGCAGATAG

> FSAC 11603

ATGCGTCTCCTGTCATTTTCTTTCTTCTTAGCGGCTCTGCTCGGGCTCGTTAATGCGGCCAGCTTCACTGAGCTAGCCGCTCAGCTCCCCGACTGTGATATACAATGTGTCCAGCAAGCGATACCGCAGTCACCATGTACATTGACGAACACAACATGTCTCTGTACAGATCAGACATTTGCAGGCTTGACGCAGGCTTGCGTGCTGAAGAACTGCACAGTCAAGGACTCACTGACATTGATGAGAGTTCAGAACCTGGCTTGCGGTGTTCCTGTCAAGAGCCAGCAGATGAAGTTCAGACTCAACGCGCTTATTGCGTGTATCCTCGCCGAGATATGTGTGATCTTGAGAATATACTCCAAACTAAAGATCTTTGGAAAGCTTGGCCCTGATGACTACGCAATTCTCATTGCAGGATGTGCTACAGTGCCTTACATCTGGCTCGCGTGCAGACTTGCAGATCTTGGCTTCGGGCTAAACATCTGGGACATCACTCCATTCGAGCGCCTGTACGAGCTGCTGAAGCTCTTCTGGATAGACCAGATCATGTACAGTGTTCACTTGTATAGCACGAAGATCTCGATTCTTTTCTTTCTCCGTGGTATTTTCACTACGTCGGAGTTTAAGAAGTTGACCGTGTCGATCGGCATCTTTGTCTGCTTGTGTGGTGCTGCTACAATGATCACCACTGCGCTTCAGTGTCTTCCAGCTTCGTACAATTGGACAAGCTGGGATGCCGAGCACAAAGGCCACTGCAATGACCTCAACGGGCAGACTTATGCTTTTGGTGGAATCAACATGGCTTGCGATATTGTCATCCTGGTTTTGCCGATTAATCACCTTTGGAAGCTACAGGTCAAGGGCCGACAGAAGATTCAGCTCTTCGTCATGTTCAGTCTGGGCATTGTTGTTACGGTGTTTAGTATCATCCGTCTGCCATTCCTGATCACGTTGGGAAAGACGACTAACCCAACCTGGGAATACGTTGAAGTCACTATCTGGTCTATCTGGGAAACGGAACTTGGCATGGTATGCGCAAGCCTCCCAGCCATCCGCCATCTCTTCAAGCATCTCTGGCCCAACGCCATGGCTACCATTGCCAGCAAGATGAGCTTCTCCAGCACAAACAAGGACTCCACACTGGACAAGAGTGCTGGAAGCTGGGGTGGTAGCCGCGGGGCTCGCTCTAAGACAGATAACAAGGAGTACTATGAGCTTGATGAGCGGAGTCTCATTGGCAAGGGGCCGGATCCTGCCACTAATGTGAGCTCCACCAACGTTCATTCGATGGCGTAG

> FSAC 13485

ATGCTCGTACCTCGAGTATTATATGCGTTTGCATTATGCGTGATACCTTTCGGCGGCTTAGCTCAAACTAATGAGCTCGATGGACTCGCCACCGATCCTTTAGCGGGCGCATCTGAGTGTGTAAGGACATGTTTCACCAAGACGAACGACACGGAAGCTTTTCAAGGCAGCCAAACAGAGGACCTATGCCAAGATAAGCCACTGTATCATGATGTTGAAATCTGTGTAAAACACGACTGCAATTTCATCGAGATACTGGACTTCAAGAACAGAACGTCACGCGCATGCGACTGGCCAATTACCGACCGTCGACAAGAAATCCGAATTAGCTGTTTGACGATAGGCATTTTGGCTATGGTCTTCTTTACTATGAGAGCCATCTCAAAGATAATTGGATTCGTGCCATATGGGCACGACGATAGTCTCATACTGGCAGCTCTGCCATTTATTATCGCATTTAATATTTTCTGCCAAGTCCTTACTTCAAACGGTCTCGGACTCGATATTTGGTTTGTGGAAGAAGATAGCATTCGCATCTTTCTCATCCTGATCTTCGCTTCCGAACTCTCATACGCTACGTCGCTCGCTCTCGTAAAGCTATCGATCTTAGCCTTTTTCCTTCGGATATTCCCCGACCAACGATTCCGCATCATTGTTCGATGGACCACCATCTTCGTTCTTGTCATGTGCCCCCTTTATCTTGCCCTCATCCTCGCTCAAAGACGGCCCCTTGATCTATTTTGGAATGGATGGAGGGACAAAAATCCACGAGGTGTTATCTTGAGTGCAAACCTCATAGGCATAACTCATGGTGCATGGAACGTTGCTTTGGACATTTGGATGATGATTCTTCCAATGACTCAACTACTCAAAATCGGGATCAAGCTAAAGAAAAAGATTGGCGTAATCGCTATGTTTGGCGTGGGACTATTTCTCACGATAGTCAGCTCCGTTCGCATTCCTAGCCTGATAGTCTTTTCAACGTCACGGAACATTACCGCCGATTCAGTCGGAATAATAATCTGGTCAAACATCGAAATCTGCGTAGGCATGATGGTGGCATGCATGCCTGGAGCACGCCAATTCGTGCGAGACGTTATACTTCGAGTTAAACGAGGAAATAATTCCGACGCGTCTAGCACTGAGAACATCTTCATCGAGAGAACGATAGAGACGATCCAAACTGATGCGGACGACACAGTCACAAGTTCTATAGTGAGACCAGAAGAAGTGCTCTTGTCAAAATGA

> FSAC 10706

ATGAAGTCCTTCTTCCTTTTCACCACTGCCATTCTCTCCTCGTCTGTTTTGGCGCAGTCGAATTCCCCGGCTGGAGGACAGGGCGGCGGCGGCAGCGTCCTTCCTGAATGCGCTCAACCTTGTGCCCTGAAGGCAATCCAAGGTAGCAGGTGCGGCTCTGAGGATCCCGGTTGCGTCTGCAAAGCTCCGGGGTTCGCTGAATCCTACGTCAGCTGCGTTGGCACCAGCTGCCGCCCTCGTGATGCCGCCGCTGCCATTTCCGCTGGCATTAGACTCTGCGAGGCTGCTGGCGTCACTATCACCGGCGTCCCGAGCGCCACGGCTGAGCACAAGGCTCCGGGCCCAACATCCGCGGCACCCGTTGTTCCCGCCCCGCCTGTCGTTCCAACTGCCCCTCCTGTTGTCACTCCAACTGCGCCCGCGCCTATTCCTGCTACACCTGTTCCCACTGCTGGTGCCAACTCCCTCAACAATGGAGTTGCGGGTATCGGAATGGCTTGGGCTTCCGTGATGTTCTTGTTCGCTCTCATGTAA

> FSAC 06761

ATGAAGGCTACTTTCTTCCTCGCTGCCGCTGGCCTCGTCGCCGCTCAGGACTTTGGTGGTCAACCCGAGTGTGCTCTCAAATGTCTCCAGGAGAACATCCCCAAGGCCGGCTGCAAGCTTGAGGACACCGCCTGCCAATGCGCCTCCGACTTCCAGGAGAAGCTCCTCCCCATCATCACCCCTTGCTTGACCAAGGCCTGCGAGGCTCAAGACCTCCTCAAGGCCCAGTCCGCTGCCGCCGAGGCCTGCAAGGAGTTCGCTAAGACCGCCGGCTCTGGCAAGCCTACAGCTACCTCTGCTGCTGCCACCACCGGCGCCGTCACCGTCAGCATCGACACCTCTGTCACCGGCTCCGCCAGCGTCCCCGCCATCTTCTCCAGCATCCCCGAGGAGTCCACCGCTGCCCCCGTTACTCCCCACCCCGGCAATGGAACCTCCACCAAGACTCACGGAGCTTCCACCCCCACTGGAACTTCTGGCTCTGGTTCCGGCAGCGAGAGTGGCAGCGCCAGCAGCGTTCCCACTGGTAACGCTGCCGCTGCCGCTGTTGCTGGCCCTGCCTTCGGTCTCCTCGCTGCTCTCGGCGCTGTCCTTGCTCTGTAA

> FSAC 08483

ATGAAAGGGCCTATTTCCATCCACCTGGTACTTTTCATGGCCTCGGTAGCCATCGCAGCCGACTCTACCTGTGCCATTGACTGCTTCCAAGGGCTCATCACGAATGGTCCTCCCGCGGGATGCAAAGAGGCAACGAACTATCTCTGCTTCTGCACAATGCCTACGCTGCAAAGCAACTTTGTGCAATGCACCGACAAGACTTGTGCCGATGAGAAAGCTGGGGCTATAGCTTGGGCAAATGAGCTCTGTGGAAAACTTGGGAAGCCTATTGATCTCGGGGGCTCTGGGGGATCTCCCAAGACTGATGAGACTACGGCCGTCGATGCCCCAACTTCCAGCGCTAGCAACGAGGTGAAGCCAACCACCGAGGCTGGGAAAACGACAGACGAGGTGCCGACTACCACTCTAGAGTCCAAGACAGAATCCAAAGTCGAGGAATCTTCAATTGAAAAGACCTCTGAGGTCACTTCTGAGTCATCAGCCGGTGAAACAACCGCAACCTCGATCAAGACCACAGTCAGCAAGCCGAAAGAAACGAACGACGCATCTGGTAGTGCAGCTGAAGCGACGGCTGAACAGACTAGCGATGACTCTGTTGTCACGGTCATTGGAACTGCCGATACCGCTACCGCTACTCCGTCTCCCACTGATGGTTCCAACGTCGCCAATTCCGCAGGACCCAACCTCTACGCTGCTGTTGGTGTTGCTGCAGTGTTTTGGCAATTACTGTAA

> FSAC 12429

ATGAAGTACTCTTTCGCTATTGCTGCTCTCGCCACCGCCGTCTCGGCTCAGACTCTTGCCGACGTCCCCAAGTGCGCTATTCCTTGCCTTGACGACGCCATCACCAGCAAGACCGACTGCAAGACCACTGATCTCACCTGTGTCTGCAAGAGCTTCGACGATGTCCGAAGCGCCGCTACCTCTTGTGTGATCACCAAGTGTGGTTCCGACGTTGCCATCAACGAGGTTCTCCCTGCTACTGAGAAGCTCTGCTCTGGCGGCTCCGGCTCCGGTTCCGCCGAGGAGTCCTCCAAGGCCGACACCACCAAGGTCGAGACCTCCACCAAGGTTGTCGTCACCACCAGCGCCGTCGAGACCACTGCTGTTGAGACCACCGCCGTCGAGACCACCTCTGTCCCTCCTGTCGTTGAGAGCAAGACCACCGCTGTTCCCCCCGTCGTTGAGACTCCCACCACCAAGTCCGAGGCTGCCGGCGGTGCCACCAGCACTCCCGCTCCCTCAGGTGTCGGCGAGAACAGCGCTGCTGGCCTCAAGGGTCTTGGCGCCATGGCCATGGCTGCCCTCATGGCCCTTGCTCTGTAA

> FSAC 13146

ATGGCCTCCCGCATTCTCCTTATTCTTGCCGCCTTTCGGCTTGTCGTCGCAGCTTCATCTACTGCCACAGAAACTACCGGCGCAACTACCTCGACCAACCCTTCTGTTATTGCTTCCGACGTTTCCTCGTTGCCAGGCTGCGGTTTGAAATGTATCTCGATAGCCGGAAAGAAGATTGGCTGCGGCGGTACTGACCTAGAATGCATGTGCTCGCACAGCGACGCGTTTGCAGACCATTTCCGGGAATGTCTGGGAAACCATTGCACTATTAAAATATTCAAAAACTTCTGGGACGTTGGCCAGCAGATTTGTGACGCGGTAGAAGGCAGTTCTAATTCTGTTGCCTTGGCATCAGCCTCAGCTGTGATTGCATCCGACATGTCCCAACAGACGGGGAGCGGTGCCGAGCGTTTGACCTACGCGGGTATCCTCGGCGCTATTACCTGGGGAGCTCTGTTGATATAG

> FSAC 08184

ATGAAGCTGTCGGTGTTCACATCCGTCCTCCTGGCTGGCCTTGTTGCCGCTCAGCAAGAGAAGTTACCAACATGCGCTCAACCATGTGTCAGCAAGTACACCACCGGTGGCGGCATCGCGGGATGCGGACAGCTCGACGTCAAGTGCATCTGCAGCAACAAGGACTTCCTCTCTGGCATCGCATGCTGTCTTGAGGATAAGTGCGATGCCGCCGGCAGGGAAACCGCCGTCAAGTACGCCAAGCAGATCTGCTCGACCGCCGGAGTGACCGATCTTCCCGACGAAGTTACATGCGACAAGAACGCCGCCTCTGGCAGCGCTTCTGGTGCCACCACTCCCACGTCCAGTGCTGGTAGCAGCCAGACCACGCCGGCGTCTGCGTCTGGCAGCGCTGGTGGCGATAGCGGCAATGCTGCTGCGCGCGAGGGCGCCGCTGGTGTCTTGGGTGTTGTCATGGCTATGCTGTTTGCCCTTTAA

> FSAC 13617

ATGAAGTCTTCTTTCCTCACCATCTTCGGCCTGGCTGCTGCCGCCGTTGCCCAGAGCTCTGATGATCTCCCCCAGTGTGGTCGTACTTGCGCCGGTAACATGGTTAGTGCCGAGAAGTCTCAGGAGCTTGGATGTGACTCCGGCGATATTGGCTGCCTGTGCACCAACCAGAACTTCATCTACGGTCTCCGTGACTGCTCTGCTGCTATCTGCAACAGTGAGCAGGCCGCCCAGGTCCTGAACTACGGTCTTGAGATTTGCCGCCGAGCTGGTGTTCAGATCACTACTGGTGCTTCTGGCGAGGTTTCGGCCACGGCTACTGGCTCTGGCGCCGTTCGAACCGTCCTTAGCACCCTGACCTCTGGTGACTCTACCATCACCTCTGCTCTCTCCACCATCAGCGGTACTGCTACTGGCGCTTCCGACGATGTTTCCGTGAGCACCTACACCAGCGTCCTCACCAACTCCGAGGGGGATGAGTTCACCACGACCGGCAAGGCTATCCTCGGCGGTGCAGTTGTCACCACTTTCACCTCTGGTGGTTCTACTATTGTGTCTACCATTGCCTCTGGAGCTGAGTCCGAGACCTCTGGAGCTGAGTCTGCCGAGGTTACCACCTTCACATCTGATGGTACTGAGATCGTCCGAACTCTCGTCACCGAGACTGCCTCCACCGACTCTGCCGCATCCGCCAAGGTTACCACATTCACCACTGATGGCACTGAGGTTGTCCGAACTCTTACCACCGTTACGAGCGGTTCTGAGTCTGAGTCGGTTTCGGAGACCGTTACTGACGCCTCCACCGCCACCGAGGGTGCTACTGATGCTAGTGGCACTGATGCTTCGGCCACCACCACCACCGGCACTGACAATGCTGCCGCTGCCCAGATGACTGGTGCCCCTGCTGGTGTCATCGCCGCCGCTGGTATTGCCATGCTCCTCCTCTAA

> FSAC 10692

ATGCAGTTCTCTACCTCTTTCCTCGTCGCTGCTCTCTTCGGTACTGCCTTTGCCATGCCCCAGGCCAACCCTACTGATTGTCCCGAGACCTCTGCTATCCCTACCTGCGGTGCTCCTTGCATCACCTCGGCCGCCTCTGCTGTTGGCTGCAGCAACATTGCTTGCCAGTGCGCCAGCTCCAGCGCCATTCAGGCTTCAGCCATCAACTGTGTCCTGGACAACTGTGGTTTCCTCGAGGCTCTGCAGGTTCAGGCTTCTGCCGCCGCTGTCTGCACTGCTTGTGCTTAG
